# Long-term cardiovascular risks and the impact of statin treatment on socioeconomic inequalities: a microsimulation model

**DOI:** 10.3399/BJGP.2023.0198

**Published:** 2024-02-20

**Authors:** Runguo Wu, Claire Williams, Junwen Zhou, Iryna Schlackow, Jonathan Emberson, Christina Reith, Anthony Keech, John Robson, Jane Armitage, Alastair Gray, John Simes, Colin Baigent, Borislava Mihaylova

**Affiliations:** Health Economics and Policy Research Unit, Wolfson Institute of Population Health, Queen Mary University of London, London, UK.; Nuffield Department of Population Health, University of Oxford, Oxford, UK.; Nuffield Department of Population Health, University of Oxford, Oxford, UK.; Nuffield Department of Population Health, University of Oxford, Oxford, UK.; Nuffield Department of Population Health and Medical Research Council Population Health Research Unit, University of Oxford, Oxford, UK.; Nuffield Department of Population Health, University of Oxford, Oxford, UK.; National Health and Medical Research Council Clinical Trials Centre, University of Sydney, Sydney, Australia.; Clinical Effectiveness Group, Wolfson Institute of Population Health, Queen Mary University of London, London, UK.; Nuffield Department of Population Health and Medical Research Council Population Health Research Unit, University of Oxford, Oxford, UK.; Nuffield Department of Population Health, University of Oxford, Oxford, UK.; National Health and Medical Research Council Clinical Trials Centre, University of Sydney, Sydney, Australia.; Nuffield Department of Population Health and Medical Research Council Population Health Research Unit, University of Oxford, Oxford, UK.; Health Economics and Policy Research Unit, Wolfson Institute of Population Health, Queen Mary University of London, London; associate professor and senior health economist, Nuffield Department of Population Health, University of Oxford, Oxford, UK.

**Keywords:** cardiovascular disease, individual patient characteristics, inequality, Markov microsimulation model, socioeconomic status, quality-adjusted life years

## Abstract

**Background:**

UK cardiovascular disease (CVD) incidence and mortality have declined in recent decades but socioeconomic inequalities persist.

**Aim:**

To present a new CVD model, and project health outcomes and the impact of guideline-recommended statin treatment across quintiles of socioeconomic deprivation in the UK.

**Design and setting:**

A lifetime microsimulation model was developed using 117 896 participants in 16 statin trials, 501 854 UK Biobank (UKB) participants, and quality-of-life data from national health surveys.

**Method:**

A CVD microsimulation model was developed using risk equations for myocardial infarction, stroke, coronary revascularisation, cancer, and vascular and non-vascular death, estimated using trial data. The authors calibrated and further developed this model in the UKB cohort, including further characteristics and a diabetes risk equation, and validated the model in UKB and Whitehall II cohorts. The model was used to predict CVD incidence, life expectancy, quality-adjusted life years (QALYs), and the impact of UK guideline-recommended statin treatment across socioeconomic deprivation quintiles.

**Results:**

Age, sex, socioeconomic deprivation, smoking, hypertension, diabetes, and cardiovascular events were key CVD risk determinants. Model-predicted event rates corresponded well to observed rates across participant categories. The model projected strong gradients in remaining life expectancy, with 4–5-year (5–8 QALYs) gaps between the least and most socioeconomically deprived quintiles. Guideline-recommended statin treatment was projected to increase QALYs, with larger gains in quintiles of higher deprivation.

**Conclusion:**

The study demonstrated the potential of guideline-recommended statin treatment to reduce socioeconomic inequalities. This CVD model is a novel resource for individualised long-term projections of health outcomes of CVD treatments.

## Introduction

Cardiovascular diseases (CVDs) are the leading cause of morbidity and mortality globally.^1^ In the UK, despite declines in age-standardised CVD mortality and morbidity over the last few decades, CVD remains a significant burden for the health system.^2^ Regional and socioeconomic inequalities persist, with slower improvements in the most deprived areas^3^^,^^4^ and increasing gaps in life expectancy at birth between the least and the most deprived quintiles from 5–7 to 6–8 years from 2001 to 2017.^3^

Disease policy models use epidemiological evidence to model key disease stages and relationships, and project the health status of individuals and populations.^5^ Unlike CVD risk equations, such as QRISK or SCORE,^6^^,^^7^ which estimate risk of a particular CVD outcome over a fixed period (typically 5 or 10 years), policy models can project long-term risks, accounting for competing risks, and enable estimation of long-term quality-of-life (QoL)-adjusted survival and costs, providing useful predictions for clinical and policy decision making. Policy models are often developed using summary data,^8^^,^^9^ which do not allow reliable assessment of outcomes in distinct patients. In recent years, however, an increasing number of CVD policy models, developed using individual-participant data (IPD), have emerged.^10^^–^^15^ These models, however, have often used older data,^13^^–^^15^ limiting their usefulness for contemporary policy analysis. Although the use of newer data to recalibrate CVD risk equations has been shown to improve their performance in contemporary populations,^16^^,^^17^ the authors of the current study are not aware of efforts to update policy models, which are structurally more complex, using newer data.

This article presents a new individual-based CVD microsimulation policy model, developed using IPD from trials and a large contemporary UK population cohort. The model was employed to assess health outcomes across UK quintiles of socioeconomic deprivation and the impact of guideline-recommended statin therapy on socioeconomic inequalities.

**Table:** How this fits in

This study presents a new cardiovascular disease microsimulation policy model that enables individualised lifetime predictions of disease risks, survival, and quality of life. The model quantified gaps at middle age of 4–5 years in life expectancy (5–8 quality-adjusted life years) across quintiles of socioeconomic deprivation in the UK. The study found that guideline-recommended statin treatment has a good potential to reduce health inequalities. Strengthening statin use would lead to larger benefits and further reductions in health inequalities.

## Method

### The CVD microsimulation policy model structure

This CVD policy model is a microsimulation model of the progression of CVD and key competing events. The model (see schematic in Supplementary Figure S1) includes seven events, namely first occurrences of myocardial infarction (MI), stroke, coronary revascularisation (including percutaneous coronary intervention and coronary artery bypass grafting), incident cancer, incident diabetes, and vascular and non-vascular death. Parametric proportional hazards risk equations inform the risk of these events. The model inputs are individuals’ characteristics of age; sex; ethnicity; body mass index (BMI); smoking status; blood pressure; lipids; haemoglobin A1C (HbA1c) and creatinine levels; previous CVD history; treated hypertension; diabetes and cancer history; mental illness; physical activity; diet quality; and socioeconomic deprivation (based on Townsend Score) (see details in Supplementary Table S1 and Supplementary Information S1). The model projects disease events and health-related QoL annually until death or 110 years of age using each individual’s characteristics. In each annual cycle, the occurrences of events are simulated in a random order. Individuals’ age and history of events are updated annually and inform the subsequent events’ risks in a time-dependent manner.

### Estimation and calibration of model risk equations

The risk equations underlying the CVD policy model were developed using Cholesterol Treatment Trialists’ (CTT) Collaboration and UK Biobank (UKB) IPD. First, Cox proportional hazards risk equations were estimated for model endpoints except incident diabetes (unavailable in CTT Collaboration data provided for this study) using 117 896 individuals from 16 randomised statin trials from the CTT Collaboration.^18^ Second, data from the 501 854 participants aged 40–70 years, recruited in the UKB between 2006 and 2010 throughout the UK, and their follow-up information until 31 March 2017^19^ was used to calibrate the risk equations and develop a *de novo* incident diabetes risk equation. Separate parametric risk equations were fitted for individuals without and with CVD history, with the exception of the incident diabetes and incident cancer equations that were fitted across all participants. Parameter uncertainty was assessed using bootstrapping.^20^ See Supplementary Information S2 for further details.

### Model validation

The model was validated by comparing the model-simulated cumulative incidences of each endpoint with the observed cumulative incidences overall and in participant categories. This included validating the UKB-calibrated model in the UKB cohort and among Whitehall II participants (*n* = 6761; 10 years’ follow-up; external validation) (see Supplementary Information S2 and S3).

### Health-related QoL

Health-related QoL associated with participant characteristics and disease histories was estimated using a linear regression model of EuroQoL-5 Dimension (EQ-5D) utility using pooled 2006, 2011, and 2017 Health Survey for England (HSE) participant data. QoL utility ranges from −0.594 for the worst health state to 1 for full health where 0 is equivalent to death and values <0 represent health states worse than death in the EQ-5D-3L used in 2006 and 2011.^21^ The EQ-5D-5L, used in HSE 2017, was mapped into EQ-5D-3L^22^ before pooling. The QoL model was integrated into the CVD model to annually predict individuals’ QoL.

### Model applications

The CVD microsimulation model was used to perform lifelong projections for all UKB participants using their baseline characteristics. The authors executed 500 microsimulations for each individual to minimise the Monte Carlo uncertainty, and probabilistic sensitivity analyses using 500 and 1000 bootstrap coefficient sets for individuals without and with CVD history at entry, respectively. The results were summarised in categories of UKB participants by CVD history, age, sex, and estimated 10-year CVD risk (QRISK 3 score).^6^

This model was used to assess the remaining life years, quality-adjusted life years (QALYs), and the effects of statin treatment, as recommended by the UK National Institute for Health and Care Excellence guidance,^8^ across quintiles of socioeconomic deprivation under the scenarios of full statin coverage and real-world use of statin treatment, as observed in UKB in 2015/2016 (Supplementary Information S2 and S4). The 10-year, 20-year, and lifetime projections of life years and QALYs for UKB participants were standardised to mid-2020 UK population distribution by sex, age, and socioeconomic quintile (based on the Index of Multiple Deprivation) (for details see Supplementary Information S2).

### Software and computing

All analyses were performed using R (version 4.2.1). The model simulation utilised Queen Mary University of London’s Apocrita High Performance Computing facility, supported by Queen Mary University of London Research-IT.^23^

### Stakeholder involvement

The project was guided by multidisciplinary project management and steering groups, including primary care and specialist clinicians, lay people, trialists, statisticians, and health economists. Three lay people were involved as members of these groups, helping refine methodology and approaches to presenting study findings.

## Results

### Summary of the CTT Collaboration and UKB data

In CTT Collaboration trials, 68 018 participants without and 49 878 with CVD history were followed over 3.9 and 4.6 years on average, respectively. In UKB, 444 576 participants without and 57 278 with CVD history were followed for 8.1 and 7.9 years on average, respectively. Their characteristics are summarised in Table 1. The details of contributing trials and the numbers of events during follow-up are summarised in Supplementary Tables S2 and S3.

**Table 1.** Baseline characteristics of study participants

**Characteristic**	**CTT Collaboration**		**UK Biobank**	

	**Without CVD history**	**With CVD history**	**Without CVD history**	**With CVD history**
***n***	68 018	49 878	444 576	57 278

**Age, years (SD)**	62.3 (9.2)	62.7 (9.1)	56.0 (8.1)	60.4 (7.0)

**Sex, male, *n* (%)**	43 972 (64.6)	39 085 (78.4)	194 996 (43.9)	33 734 (58.9)

**Ethnicity, *n* (%)**				
White	49 170 (72.3)	38 901 (78.0)	420 409 (94.6)	54 488 (95.1)
Black	6110 (9.0)	1226 (2.5)	7268 (1.6)	770 (1.3)
South Asian	NA	NA	6946 (1.6)	1053 (1.8)
Other^a^	12 738 (18.7)	9751 (19.5)	9953 (2.2)	967 (1.7)

**Smoking status, *n* (%)**				
Non-smoker	54 145 (79.6)^b^	40 324 (80.8)^b^	250 261 (56.3)	25 137 (43.9)
Ex-smoker	NA	NA	148 312 (33.4)	25 211 (44.0)
Current smoker	13 873 (20.4)	9554 (19.2)	46 003 (10.3)	6930 (12.1)

**BMI (kg/m^2^), *n* (%)**				
<18.5	670 (1.0)	251 (0.5)	2370 (0.5)	256 (0.4)
≥18.5–<25	19 963 (29.3)	15 023 (30.1)	149 300 (33.6)	13 415 (23.4)
≥25–<30	28 598 (42.0)	23 711 (47.5)	189 650 (42.7)	24 241 (42.3)
≥30–<35	13 532 (19.9)	8487 (17.0)	74 714 (16.8)	13 222 (23.1)
≥35–<40	3708 (5.5)	1806 (3.6)	20 662 (4.6)	4328 (7.6)
≥40	1547 (2.3)	600 (1.2)	7880 (1.8)	1816 (3.2)

**LDL cholesterol, mmol/L, mean (SD)**	3.5 (0.89)	3.8 (0.85)	3.6 (0.82)	3.1 (0.87)

**HDL cholesterol, mmol/L, mean (SD)**	1.3 (0.38)	1.1 (0.31)	1.5 (0.37)	1.3 (0.36)

**HbA1c, mmol/mol, mean (SD)**	NA	NA	35.8 (6.2)	38.6 (8.7)

**Creatinine, umol/L, mean (SD)**	92 (23.8)	98 (22.7)	71 (15.1)	77 (19.8)

**Systolic blood pressure, mmHg, mean (SD)**	142 (20.3)	139 (22.1)	138 (18.6)	139 (18.9)

**Diastolic blood pressure, mmHg, mean (SD)**	83 (11.2)	81 (11.3)	82 (10.1)	81 (10.5)

**On hypertension treatment, *n* (%)**	35 478 (52.2)	25 472 (51.1)	71 930 (16.2)	26 184 (45.7)

**Prior diabetes (any), *n* (%)**	15 131 (22.2)	7949 (15.9)	21 567 (4.9)	8171 (14.3)
Prior type 1 diabetes, *n* (%)	NA	NA	2741 (0.6)	1479 (2.6)

**Prior cancer, *n* (%)**	32 (0.1)	25 (0.1)	32 713 (7.4)	5861 (10.2)

**Prior CVD, *n* (%)**				
MI only	NA	24 866 (49.9)	NA	2071 (3.6)
Peripheral arterial disease only	NA	3186 (6.4)	NA	6806 (11.9)
Stroke only	NA	3875 (7.8)	NA	5137 (9.0)
Other coronary heart disease only^c^	NA	9631 (19.3)	NA	28 973 (50.6)
≥2	NA	8320 (16.7)	NA	14 291 (25.0)

**Townsend deprivation score,^d^ *n* (%)**				
Quintile 1 (least deprived)	NA	NA	166 141 (37.4)	18 960 (33.1)
Quintile 2	NA	NA	89 397 (20.1)	10 957 (19.1)
Quintile 3	NA	NA	72 626 (16.3)	9034 (15.8)
Quintile 4	NA	NA	64 448 (14.5)	9178 (16.0)
Quintile 5	NA	NA	51 964 (11.7)	9149 (16.0)

**Physical activity, *n* (%)**				
High level	NA	NA	145 206 (32.7)	16 780 (29.3)
Moderate level	NA	NA	146 156 (32.9)	17 679 (30.9)
Low level	NA	NA	65 932 (14.8)	10 105 (17.6)
Missing	NA	NA	87 282 (19.6)	12 714 (22.2)

**History of severe mental illness, *n* (%)**	NA	NA	36 087 (8.1)	6324 (11.0)

**Unhealthy diet (including uncertain), *n* (%)**	NA	NA	158 569 (35.7)	21 705 (37.9)

a *Other ethnicity includes Chinese, Mixed, White and Black Caribbean, White and Black African, White and Asian, any other mixed background, and other ethnic group.*

b *Includes ex-smokers.*

c *Other coronary heart disease includes acute rheumatic fever, chronic rheumatic heart diseases, hypertensive heart disease, angina pectoris, other acute ischaemic heart disease, chronic ischaemic heart disease, pulmonary heart disease, and other form of heart disease.*

d *Townsend Score used to categorise UK Biobank participants into quintiles of socioeconomic deprivation using national cut-off values. BMI = body mass index. CTT = Cholesterol Treatment Trialists. CVD = cardiovascular disease. HbA1c = haemoglobin A1C. HDL = high-density lipoprotein. LDL = low-density lipoprotein. MI = myocardial infarction. NA = not available. SD = standard deviation.*

### Risk equations

**Figure 1.**
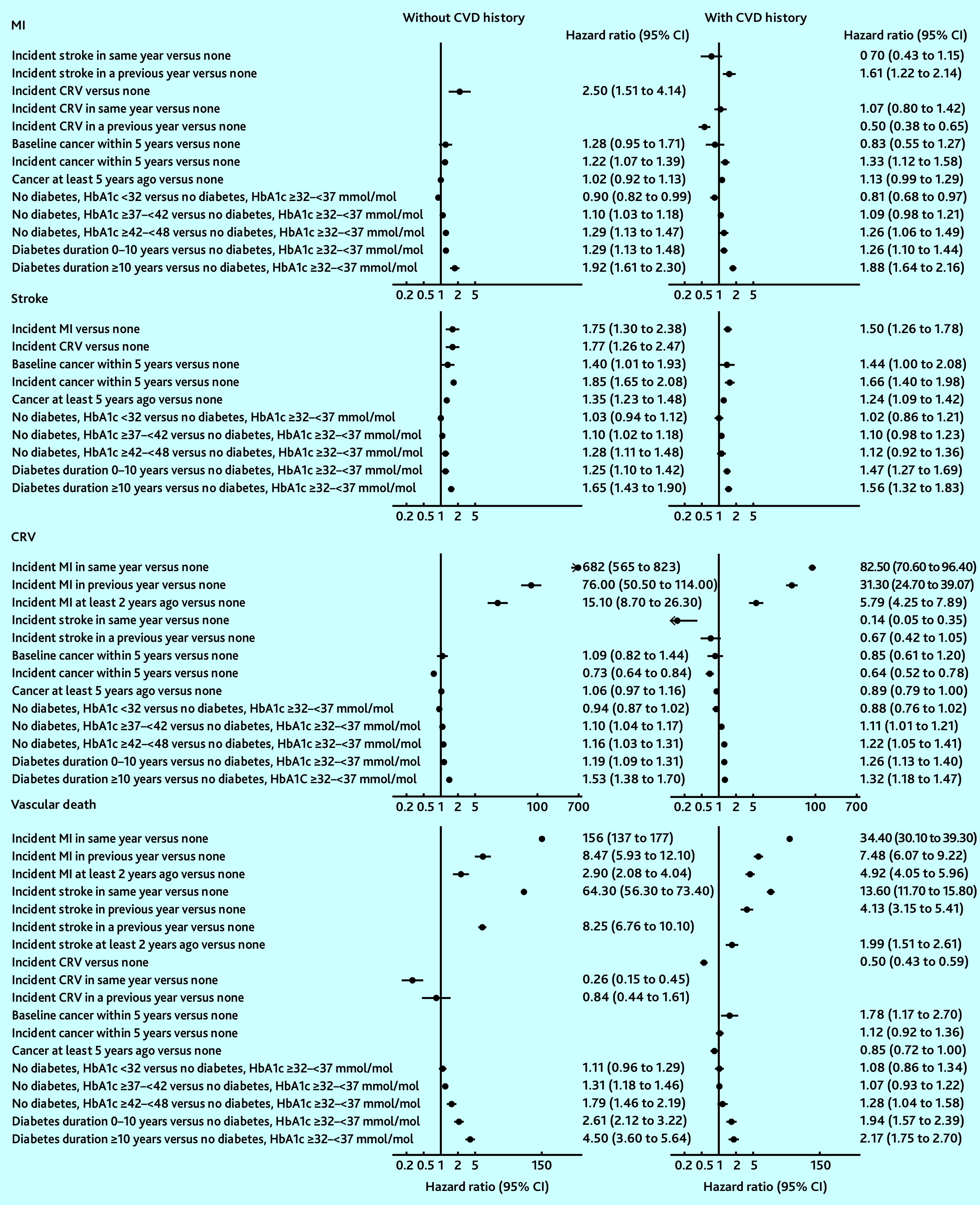
Risk of vascular endpoints associated with disease-event histories. Adjusted for other individual characteristics at entry and current age. See Supplementary Tables S4–S6 for full details of the risk equations. CRV = coronary revascularisation. CVD = cardiovascular disease. HbA1c = haemoglobin A1C. MI = myocardial infarction.

MI was strongly associated with an increased risk of stroke, and MI and stroke were associated with an increased risk of vascular death, with increases greatest in the years of these events. Coronary revascularisation was associated with a reduced risk of vascular death. Duration since diabetes diagnosis was associated with increased risks of all cardiovascular events and, in those without diagnosed diabetes, higher HbA1c levels were associated with increased risks of all cardiovascular events. These patterns were similar between people without and with CVD history although magnitudes differed (Figure 1 and Supplementary Tables S4–S6).

Age, male sex, smoking, treated hypertension, unhealthy diet, and lower physical activity were strongly associated with an increased risk of cardiovascular events. Greater socioeconomic deprivation was associated with a higher risk of stroke, incident diabetes, and vascular and non-vascular death (Supplementary Tables S4–S6).

### Model validation

**Figure 2.**
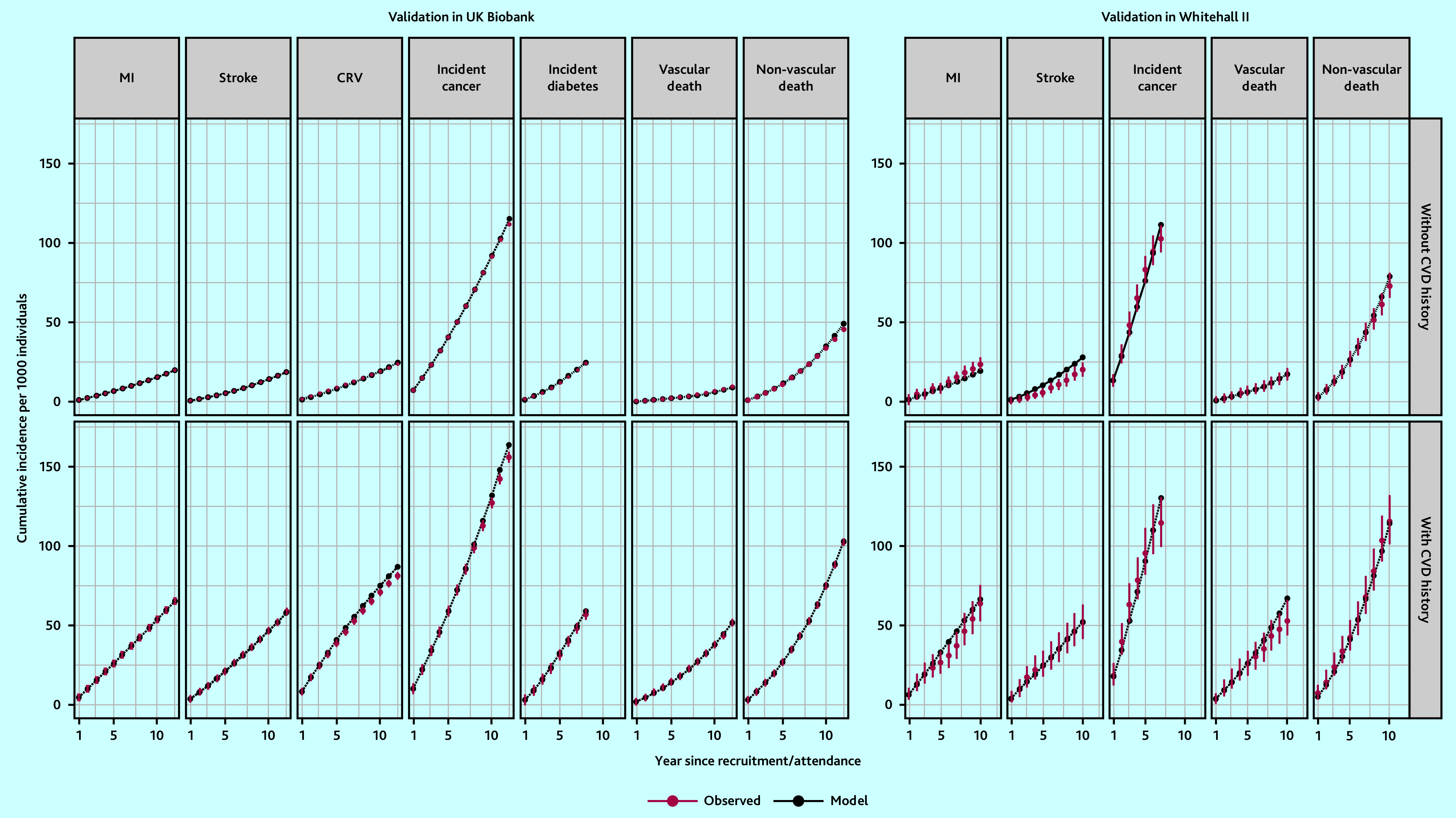
Validation of the CVD model in UK Biobank and Whitehall II Phase 9 participants. Validation covers 12 years in UK Biobank (8 years for incidence diabetes because of stopping follow-up earlier) and 10 years in Whitehall II Phase 9 data (7 years for incidence cancer because of stopping follow-up earlier). CRV = coronary revascularisation. CVD = cardiovascular disease. MI = myocardial infarction.

In the internal validation of the model based on CTT Collaboration risk equations, the model-predicted cumulative incidence rates closely matched the observed rates (Supplementary Figure S2). External validation of the model based on CTT Collaboration risk equations in the UKB cohort indicated the need for calibration to improve the accuracy of predictions. After calibration, there was good correspondence between cumulative incidence rates predicted by the model and the observed rates in the UKB for all endpoints across follow-up years in categories of participants (Figure 2 and Supplementary Figures S3 and S4). The UKB-calibrated model demonstrated a good overall performance in the external Whitehall II cohort (Figure 2).

### Effects of CVD events on QoL

CVD was a key determinant of QoL. MI was associated with a decrement in EQ-5D utility of 0.10 (95% confidence interval [CI] = 0.03 to 0.16) in the year of event and 0.07 (95% CI = 0.04 to 0.10) in subsequent years. Stroke was associated with a decrement of 0.09 (95% CI = 0.04 to 0.13) in the year of event and 0.13 (95% CI = 0.11 to 0.16) in subsequent years. Diabetes was associated with a decrement of 0.04 (95% CI = 0.03 to 0.06) in the first 10 years from diagnosis and 0.08 (95% CI = 0.06 to 0.10) in subsequent years. Cancer affecting daily life was associated with a decrement in QoL of 0.13 (95% CI = 0.11 to 0.14) (Supplementary Table S7). For use in the CVD microsimulation model, the cancer-related utility decrement was revised to 0.03 informed by the literature^24^^–^^26^ to reflect any cancer history.

### Long-term projections of survival and QALYs

**Figure 3.**
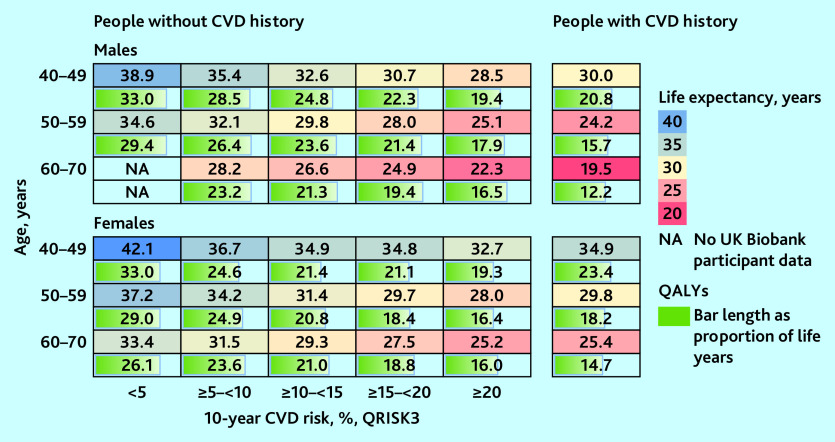
Predicted remaining life expectancy (years) and QALYs for UK Biobank participants. Predicted outcomes presented by CVD history, sex, age, and, for people without CVD history, by 10-year CVD risk (QRISK3). CVD = cardiovascular disease. QALY = quality-adjusted life year. NA = not available.

The differences in CVD risk between individuals of different age, sex, and cardiovascular risk were reflected in their model-predicted survival and QALYs (Figure 3). For individuals in the same age and sex category, shorter life expectancy and fewer QALYs were predicted for people with CVD history or higher 10-year CVD risk. Males had shorter life expectancy but more QALYs as a proportion of their life expectancy than females. The projected remaining life expectancy (from age at entry) ranged between 19.5 (95% CI = 18.7 to 20.4) and 38.9 (95% CI = 37.3 to 40.2) years (12.2 [95% CI = 11.7 to 12.8] to 33.0 [95% CI = 31.8 to 34.0] QALYs) for males, and 25.2 (95% CI = 24.2 to 25.9) and 42.1 (95% CI = 40.3 to 43.7) (14.7 [95% CI = 14.0 to 15.5] to 33.0 years (95% CI = 31.8 to 34.0] QALYs) for females. Being in the highest 10-year CVD risk category (≥20%) or having CVD history was associated with up to 10 years’ lower life expectancy and 14 less QALYs, compared with those in the same age group with the lowest risk (see summary of parameter uncertainty in Supplementary Tables S8–S10 and the events’ accumulation in Supplementary Figure S5).

### Health outcomes across quintiles of socioeconomic deprivation

**Figure 4.**
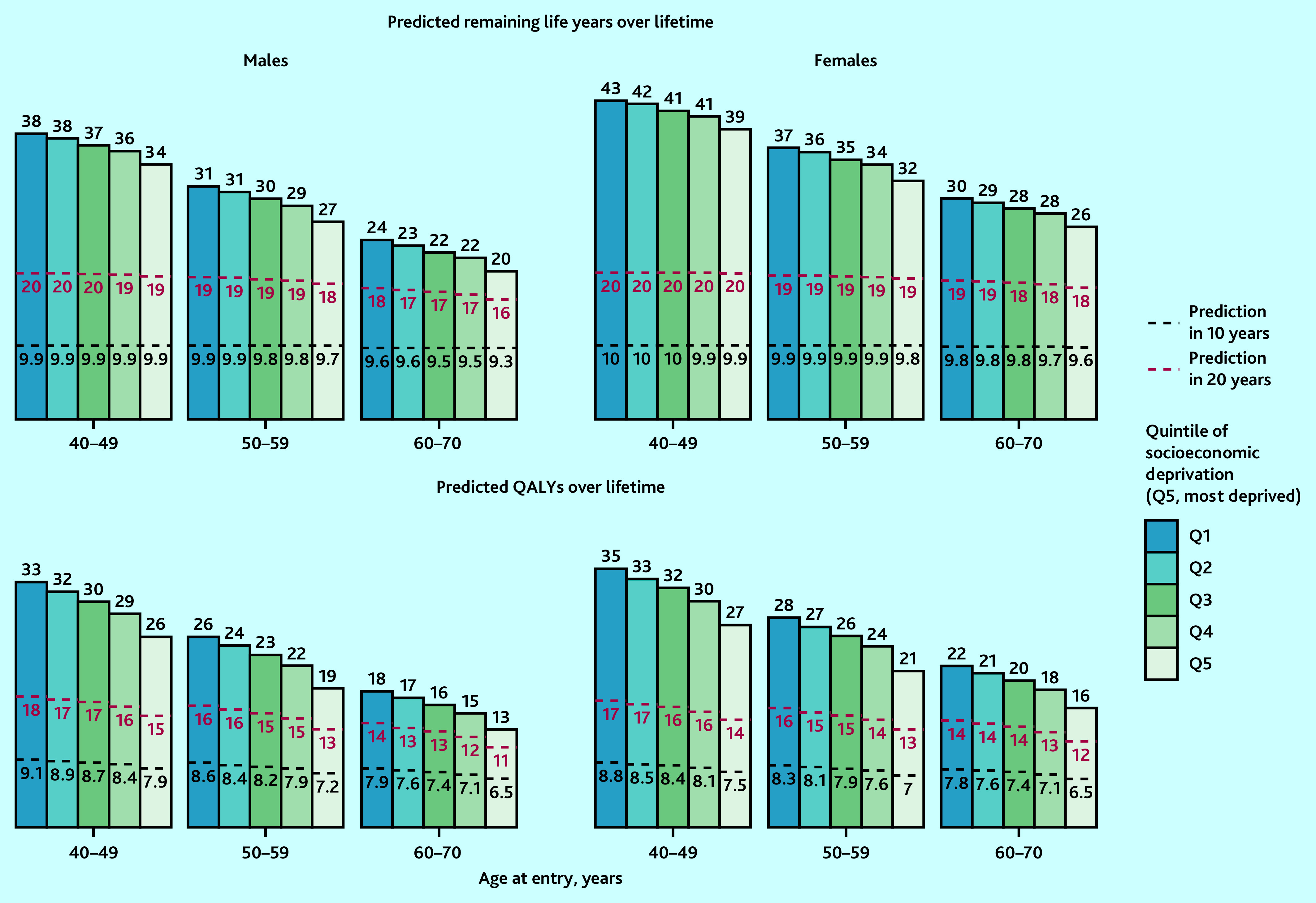
Predicted life years and QALYs in 10 years, 20 years, and over lifetime, by sex, age, and quintile of socioeconomic deprivation in the UK. Predicted remaining life years and QALYs by age, sex, and socioeconomic deprivation quintiles (using Townsend Score) at entry into UK Biobank were standardised to mid-2020 UK population distribution by age, sex, and quintile of socioeconomic deprivation (using Index of Multiple Deprivation quintiles). The bars represent remaining life years or QALYs over lifetime and the areas under the black-dotted lines and red-dotted lines represent QALYs in 10 years and QALYs in 20 years, respectively. CVD = cardiovascular disease. QALY = quality-adjusted life year.

There were moderate gradients at 10 years in the predicted years of life and QALYs across the quintiles of socioeconomic deprivation (Figure 4). Over a longer duration, the gradients in the predicted survival and QALYs increased. Over a lifetime, individuals in the most socioeconomically deprived quintile had 4–5 years’ shorter life expectancy (5–8 less QALYs) than those in the least deprived quintile.

### Benefits from statin therapy across quintiles of socioeconomic deprivation

**Figure 5.**
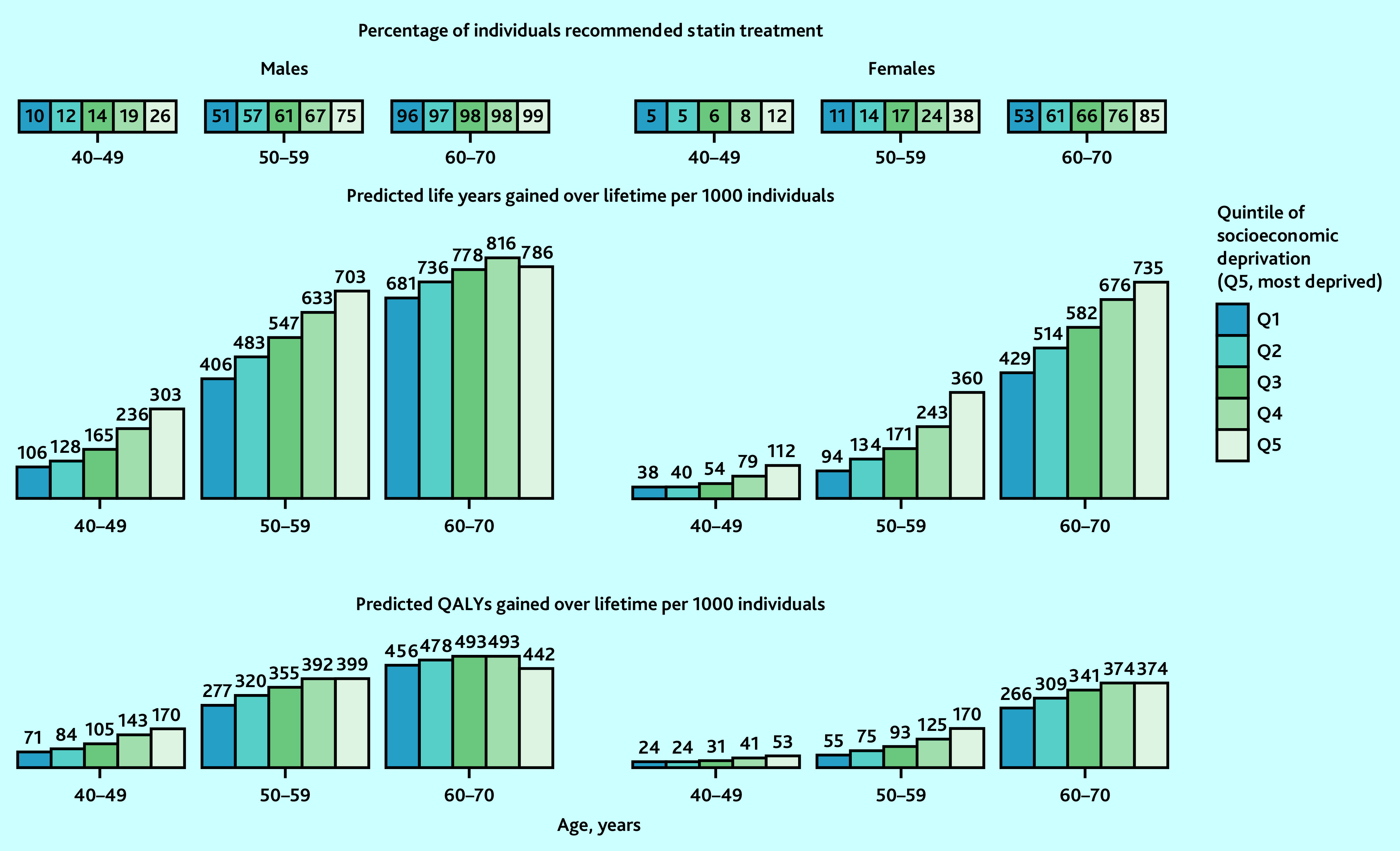
Predicted lifetime benefit from UK guideline-recommended statin therapy, by sex, age, and quintile of socioeconomic deprivation in UK. Predicted life years and QALYs gained with full implementation of UK National Institute for Health and Care Excellence guideline-recommended statin therapy at entry into UK Biobank were standardised to mid-2020 UK population distribution by age, sex, and quintile of socioeconomic deprivation (using Index of Multiple Deprivation quintiles). Atorvastatin 20 mg/day was used for individuals without cardiovascular (CVD) history but with a 10-year CVD risk ≥10% and/or type 1 diabetes, an estimated glomerular filtration rate (eGFR) <60 mL/min/1.73^2^, or albuminuria; and atorvastatin 80 mg/day for individuals with CVD history (20 mg in those with eGFR <60 mL/min/1.73^2^). QALY = quality-adjusted life year.

UKB participants in more socioeconomically deprived quintiles were more likely to be at higher CVD risk and, therefore, meet statin treatment criteria, and gained more benefit. For instance, compared with no use of statin therapy, if all people in their 50s recommended statin therapy were initiating and taking it over their lifetime, 703 and 360 life years (399 and 170 QALYs) per 1000 males and females were projected to be gained in the most deprived quintile, respectively, compared with 406 and 94 life years (277 and 55 QALYs) projected to be gained in the least deprived quintile (Figure 5).

In scenario analysis, the real-world use of statin treatment among eligible participants ranged from about 40% in the least deprived to 45% in the most deprived quintile and, although treatment benefits were proportionately reduced, larger benefits were projected in quintiles with higher socioeconomic deprivation (Supplementary Figure S6).

### Model web interface and prediction for a typical individual

The CVD microsimulation model interface and user guide are available at https://livedataoxford.shinyapps.io/shiny_ctt_ukb_model. The model interface enables users to project outcome for one or a group of patients. To illustrate its use, the model predicted 5.2% 10-year and 40% lifetime cumulative incidence of major vascular event (MI, stroke, coronary revascularisation, or vascular death), 28 years’ further lifespan, and 23 QALYs over the lifetime for a 60-year-old White male, non-smoker, overweight (BMI 25–30 kg/m^2^), in quintile 3 of socioeconomic deprivation, with moderate physical activities, a healthy diet, a low-density lipoprotein cholesterol of 3.6 mmol/L, a high-density lipoprotein cholesterol of 1 mmol/L, creatinine of 82 umol/L, blood pressure of 140/80 mmHg, HbA1c of 40 mmol/mol, not on antihypertensive or statin treatment, without histories of CVD, severe mental illness, cancer, or diabetes, and with 12.6% estimated 10-year CVD risk (QRISK3).

For UKB participants without CVD at entry, Supplementary Figure S7 presents model-predicted 10-year risks of major vascular event against QRISK3 10-year CVD risks.

## Discussion

### Summary

This study presents a new CVD microsimulation policy model that enables individualised lifetime predictions of disease risks, survival, and QoL. The model demonstrated good predictive accuracy across diverse patient categories of the UK population. The model quantified gaps at middle age of 4–5 years in life expectancy (corresponding to 5–8 QALYs) across quintiles of socioeconomic deprivation in the UK and demonstrated good potential of guideline-recommended statin treatment to reduce these gaps.

### Strengths and limitations

The main strengths of this CVD model are in its use of rich contemporary IPD, including a larger set of characteristics and disease endpoints than other previous studies. First, the model has the advantage of reflecting contemporary CVD trends in the UK, being derived from a large current UK population cohort. Second, it includes a rich set of individual characteristics of high policy interest, including socioeconomic deprivation, physical activity, and diet quality. Third, the microsimulation framework can track individuals’ history and multiple interacting comorbidities.^27^ Fourth, the model includes incident diabetes and cancer as key competing disease risks to better reflect overall health and enable inclusion of a broader range of effects. Finally, the model can inform assessments of the net effects and cost-effectiveness of therapies.

The CVD model has some limitations. The UKB is a cohort of people healthier than the general UK population, with underrepresentation of those in more socioeconomically deprived categories.^28^ Notwithstanding this, the model projections for different individuals using their characteristics are likely generalisable to similar individuals in the general population. To enhance generalisability of the current findings, the results were standardised to the 2020 UK population by age, sex, and socioeconomic deprivation. Moreover, the oldest individuals during UKB follow-up were in their 80s and further research is needed to assess the model performance among older people.

### Comparison with existing literature

The inequalities in health outcomes associated with socioeconomic deprivation at age 40–70 years reported in the present study are expectedly smaller than the previous estimates of 6–8 years estimated at birth,^3^ but they echo studies suggesting that socioeconomic deprivation remains an important health determinant.^29^^–^^31^ The current study also quantified and reported larger gradient in inequalities when QoL was considered in addition to individuals’ survival. Similar to recent studies,^32^^–^^35^ the authors note that the use of statin treatment was not worse among those in the more socioeconomically disadvantaged categories.

### Implications for practice

The CVD microsimulation model could help inform strategies for CVD management, including assessments of their cost-effectiveness across UK population categories and the impact on health inequalities.

This study found that guideline-recommended statin treatment has good potential to reduce inequalities in health across socioeconomic status, although the reductions are larger for differences in life expectancy than for QALYs, underlining the need for multifaceted action to tackle health disparities. Strengthening statin use would lead to larger benefits and larger reductions in health inequalities.

## Data

Requests for individual patient data from trials contributing into the Cholesterol Treatment Trialists’ (CTT) Collaboration should be made directly to the data custodians of each trial (see CTT Collaboration data policy at https://www.cttcollaboration.org). Other data underlining this work may be obtained from third parties (UK Biobank: https://www.ukbiobank.ac.uk; and Whitehall II study: www.ucl.ac.uk/epidemiology-health-care/research/epidemiology-and-public-health/research/whitehall-ii) and are not publicly available. Researchers can apply to use the UK Biobank resource and Whitehall II study data. The R code of the CVD microsimulation model (License: MIT License) is available at http://www.herc.ox.ac.uk/downloads/supportingmaterial and from https://github.com/RunguoWu/UK_CVD_model.
