# Supplementary material for: Long-term cardiovascular risks and statin treatment impact on socioeconomic inequalities: microsimulation model
Source: Br J Gen Pract. Author manuscript; Available in PMC 2024 Mar 5. (PMC10904120; doi:10.3399/BJGP.2023.0198)
Supplement: Supplementary information [file EMS188946-supplement-Supplementary_information.pdf]

Long-term cardiovascular risks and statin treatment impact on socioeconomic inequalities: microsimulation model

Supplementary material

Contents

Supplementary Table S1: ICD codes, OPCS codes and UKB study codes used to define event endpoints and participant disease histories at entry into UKB ... 3
Supplementary Table S2: Baseline characteristics of participants, by trial ... 7
Supplementary Table S3: Number of study participants with events during follow-up ... 8
Supplementary Table S4: Parametric proportional hazards risk equations: myocardial infarction and stroke ... 9
Supplementary Table S5: Parametric proportional hazards risk equations: coronary revascularisation, incident cancer and incident diabetes ... 12
Supplementary Table S6: Parametric proportional hazards risk equations: vascular death and nonvascular death ... 15
Supplementary Table S7: The health-related quality of life (QoL) model ... 18
Supplementary Table S8: Lifelong simulation results (95% CI), men without CVD history ... 19
Supplementary Table S9: Lifelong simulation results (95% CI), women without CVD history ... 20
Supplementary Table S10: Lifelong simulation results (95% CI), men and women with CVD history ... 21
Supplementary Figure S1: Development and structure of the CVD microsimulation model ... 22
Supplementary Figure S2: Internal validation of the first-stage CVD model based on CTT data ... 23
Supplementary Figure S3: Validation of the calibrated CVD microsimulation model in the UKB cohort by sex, age, ethnicity and history of diabetes at entry ... 24
Supplementary Figure S4: Validation of the calibrated CVD microsimulation model in the UKB cohort by quintile of socioeconomic deprivation ... 25
Supplementary Figure S5: Accumulation of cardiovascular events of UKB participants over time in the model in following 30 years ... 26
Supplementary Figure S6: Predicted lifetime benefit from UK guideline-recommended statin therapy, by sex, age and quintile of socioeconomic deprivation in UK, with real-world statin use among eligible people ... 27
Supplementary Figure S7: Model predicted 10-year risks of major vascular event versus QRISK 3 10-year CVD risk at baseline for UKB participant without previous cardiovascular disease at entry, by sex ... 28
Supplementary methods 1: Data preparation ... 29
  The methods of handling missing data in UK Biobank ... 29
  QRISK3 derivation in UK Biobank ... 30
  Quintiles of Townsend score ... 30
  Definition of physical activity levels ... 30
  Definition of severe mental illness ... 31
  Definition of unhealthy diet ... 31
  Definition of diabetes refined using primary care data ... 31
Supplementary methods 2: The modelling procedure ... 33
  The initial estimation of model risk equations ... 33

Calibration of model risk equations and further development of the model 34
Model simulation and summary of results 35
Standardising model projections to UK population distribution 35
Supplementary methods 3: Description of Whitehall II data 37
Supplementary methods 4: Statin guideline recommendations and effects of statin treatment 39
Statin effects on cardiovascular endpoints 39
Adverse effects of statin treatment 40
Real-world statin use scenario.. 40
Supplementary references 42

Supplementary Table S1: ICD codes, OPCS codes and UKB study codes used to define event endpoints and participant disease histories at entry into UKB

Code	Code description	Code system	Used in data sources
Myocardial infarction			
1075	Heart attack/myocardial infarction	UKB non-cancer illness codes	Baseline Interview and UKB algorithms
I21	Acute myocardial infarction (all subclasses)	ICD-10	Hospital inpatient, Death register and UKB algorithms
I22	Subsequent myocardial infarction (all subclasses)		
I23	Certain current complications following acute myocardial infarction (all subclasses)		
I241	Dressler's syndrome		
I252*	Old myocardial infarction		
410	Acute myocardial infarction (all subclasses)	ICD-9	Hospital inpatient and UKB algorithms
4119	Other acute and subacute forms of ischaemic heart disease		
4129*	Old myocardial infarction		
4298	Other ill-defined descriptions and complications of heart disease		
Stroke			
1081	Stroke	UKB non-cancer illness codes	Baseline Interview and UKB algorithms
1086	Subarachnoid haemorrhage		
1491	Brain haemorrhage		
1583	Ischaemic stroke		
1083	subdural haemorrhage/haematoma		
I60	Subarachnoid haemorrhage (all subclasses)	ICD-10	Hospital inpatient, Death register and UKB algorithms
I61	Intracerebral haemorrhage (all subclasses)		
I62	Other nontraumatic intracranial haemorrhage (all subclasses)		
I63	Cerebral infarction (all subclasses)		
I64	Stroke, not specified as haemorrhage or infarction		
430	Subarachnoid haemorrhage	ICD-9	Hospital inpatient and UKB algorithms
431	Intracerebral haemorrhage		
432	Other and unspecified intracranial haemorrhage		
434	Occlusion of cerebral arteries		
436	Acute but ill-defined cerebrovascular disease		
Coronary revascularisation†			
K40	Saphenous vein graft replacement of coronary artery (all subclasses)	OPCS-4	Hospital inpatient
K41	Other autograft replacement of coronary artery (all subclasses)		
K42	Allograft replacement of coronary artery (all subclasses)		
K43	Prosthetic replacement of coronary artery (all subclasses)		
K44	Other replacement of coronary artery (all subclasses)		
K45	Connection of thoracic artery to coronary artery (all subclasses)		
K46	Other bypass of coronary artery (all subclasses)		
K49	Transluminal balloon angioplasty of coronary artery (all subclasses)		
K75	Percutaneous transluminal balloon angioplasty and insertion of stent into coronary artery (all subclasses)		
K76	Transluminal operations on cardiac conduit (all subclasses)		
K501	Percutaneous transluminal laser coronary angioplasty		
K504	Percutaneous transluminal atherectomy of coronary artery		
Cancer			
All codes (except	All cancers except non-melanoma skin cancers	UKB cancer codes	Baseline Interview

1060-62, 1073)			
C category (except C44)	All categories starting with letter C, i.e. malignant neoplasms, except non-melanoma skin cancers	ICD-10	Hospital inpatient, Death register and cancer register
140-208	malignant neoplasms, except non-melanoma skin cancers (all subclasses except 173)	ICD-9	Hospital inpatient, Death register and cancer register
	Diabetes‡		
1220	Diabetes	UKB non-cancer illness codes	Hospital inpatient and UKB algorithms
1222	Type 1 diabetes		
1223	Type 2 diabetes		
E10	Insulin-dependent diabetes mellitus (all subclasses)	ICD-10	Hospital inpatient, Death register and UKB algorithms
E11	Non-insulin-dependent diabetes mellitus (all subclasses)		
E12	Malnutrition-related diabetes mellitus (all subclasses)		
E13	Other specified diabetes mellitus (all subclasses)		
E14	Unspecified diabetes mellitus (all subclasses)		
250	Diabetes mellitus	ICD-9	Hospital inpatient and UKB algorithms
2500	Diabetes mellitus without mention of complication (all subclasses)		
2501	Diabetes with ketoacidosis (all subclasses)		
2502	Diabetes with coma (all subclasses)		
2503	Diabetes with renal manifestations		
2504	Diabetes with ophthalmic manifestations		
2505	Diabetes with neurological manifestations		
2509	Diabetes with unspecified complications (all subclasses)		
C10	Diabetes mellitus	Read 2	Primary care data
	Vascular death		
I category	Diseases of the circulatory system as the underlying cause	ICD-10	Death register
R category	Symptoms, signs and abnormal clinical and laboratory findings, not elsewhere classified as the underlying cause		
W19	Unspecified fall as the underlying cause + Diseases of the circulatory system as the secondary cause		
Y832	Surgical operation with anastomosis, bypass or graft + Diseases of the circulatory system as the secondary cause		
Y835	Amputation of limb(s) + Diseases of the circulatory system as the secondary cause		
	Hypertension (at entry into UK Biobank)		
1065	Hypertension	UKB non-cancer illness codes	Baseline Interview and UKB algorithms
1072	Essential hypertension		
I10	Essential (primary) hypertension	ICD-10	UKB algorithms
I11	Hypertensive heart disease		
I12	Hypertensive renal disease		
I13	Hypertensive heart and renal disease		
I15	Secondary hypertension		
	Other coronary heart diseases (at entry into UK Biobank)		
1074	Angina	UKB non-cancer illness codes	Baseline Interview and UKB algorithms
1076	Heart failure/pulmonary odema		
I01	Rheumatic fever with heart involvement	ICD-10	UKB algorithms

I02	Rheumatic chorea		
I05-I09	Chronic rheumatic heart diseases		
I11	Hypertensive heart disease		
I13	Hypertensive heart and renal disease		
I20	Angina pectoris		
I24§	Other acute ischaemic heart diseases		
I26-I28	Pulmonary heart disease and diseases of pulmonary circulation		
I30-52	Other forms of heart disease		
I25	Chronic ischaemic heart disease (excluding I252 old MI)		Hospital inpatient
414	Other forms of chronic ischaemic heart disease	ICD-9	
Peripheral artery diseases (at entry into UK Biobank)			
1067	Peripheral vascular disease	UKB non-cancer illness codes	Baseline Interview
1087	leg claudication/intermittent claudication	Operation code	
1088	arterial embolism		
1492	aortic aneurysm		
1591	aortic aneurysm rupture		
1592	aortic dissection		
1071	other arterial surgery/revascularisation procedures		
1102	fem-pop bypass/leg artery bypass		
1103	leg artery aneurysm repair		
1555	femoral/popliteal/iliac aneurysm repair		
1104	aortic aneurysm/repair or stent		
1105	carotid artery surgery/endarterectomy		
1107	non-coronary artery angioplasty +/- stent		
1108	leg artery angioplasty +/- stent		
1109	carotid artery angioplasty +/- stent		
1110	renal artery angioplasty +/- stent		
1440	amputation of leg		
1441	amputation of foot		
1442	amputation of toe		
I71	aortic aneurysm and dissection	ICD-10	UKB algorithms
I72	other aneurysm		
I73	other peripheral vascular diseases		
I74	arterial embolism and thrombosis		
I77	other disorders of arteries and arterioles		
L16	Extra-anatomic bypass of aorta	OPCS-4	Hospital inpatient
L18	Emergency replacement of aneurysmal segment of aorta		
L19	Other replacement of aneurysmal segment of aorta		
L20	Other emergency bypass of segment of aorta		
L21	Other bypass of segment of aorta		
L22	Attention to prosthesis of aorta		
L23	Plastic repair of aorta		
L25	Other open operations on aorta		
L26	Transluminal operations on aorta		
L27	Transluminal insertion of stent graft for aneurysmal segment of aorta		
L28	Transluminal operations on aneurysmal segment of aorta		
L29	Reconstruction of carotid artery		
L30	Other open operations on carotid artery		
L31	Transluminal operations on carotid artery		
L37	Reconstruction of subclavian artery		
L38	Other open operations on subclavian artery		
L39	Transluminal operations on subclavian artery		
L41	Reconstruction of renal artery		

L42	Other open operations on renal artery		
L43	Transluminal operations on renal artery		
L45	Reconstruction of other visceral branch of abdominal aorta		
L46	Other open operations on other visceral branch of abdominal aorta		
L47	Transluminal operations on other visceral branch of abdominal aorta		
L48	Emergency replacement of aneurysmal iliac artery		
L49	Other replacement of aneurysmal iliac artery		
L50	Other emergency bypass of iliac artery		
L51	Other bypass of iliac artery		
L52	Reconstruction of iliac artery		
L53	Other open operations on iliac artery		
L54	Transluminal operations on iliac artery		
L56	Emergency replacement of aneurysmal femoral artery		
L57	Other replacement of aneurysmal femoral artery		
L58	Other emergency bypass of femoral artery		
L59	Other bypass of femoral artery		
L60	Reconstruction of femoral artery		
L62	Other open operations on femoral artery		
L63	Transluminal operations on femoral artery		
L65	Revision of reconstruction of artery		
L66	Other therapeutic transluminal operations on artery		
L67	Excision of other artery		
L68	Repair of other artery		
L70	Other open operations on other artery		
L71	Therapeutic transluminal operations on other artery		
L74	Arteriovenous shunt		
L75	Other arteriovenous operations		
L76	Endovascular placement of stent		
L89	Other endovascular placement of stent		
881	Endarterectomy	OPCS-3	
884	Repair of artery		
8841	Repair of artery : by-pass graft (vein)		
8843	Repair of artery : repair by prosthesis		
8845	Repair of artery : suture of artery		
885	Ligation of artery, not elsewhere classified		
8852	Ligation of artery, not elsewhere classified : control of aneurysm		
8853	Ligation of artery, not elsewhere classified : post operative		
8891	Other operations on arteries : production of arterio-venous fistula		
8892	Other operations on arteries : revision of previous vascular operation		

*only included for baseline MI. †OPCS-3 codes all have an associated data earlier than recruitment, while we only use incident coronary revascularisation after recruitment. ‡these diabetes codes were mapped into the ICD-10 code system in UKB first occurrence algorithms, which we used to generate diabetes variables. We also used medication information in primary care data for identification of diabetes and its types. See definition of diabetes refined using primary care data in Supplementary Methods 1. §I241 Dressler's syndrome was counted as MI but all come with other CHD codes. ICD, International Classification of Diseases; OPCS, Office of Population Censuses and Surveys, as the Classification of Surgical Operations and Procedures.(1)

Supplementary Table S2: Baseline characteristics of participants, by trial

Trial name	n	Age	Male sex	Smoker	Ethnicity - White	Ethnicity - Black	Ethnicity - Other or n/a	On hypertension treatment	History of Diabetes	Prior CVD overall
		mean(sd)	n (%)	n (%)	n (%)	n (%)	n (%)	n (%)	n (%)	n (%)
SSSS	4444	58.62 (7.07)	3617 (81.4)	1138 (25.6)	4443 (100.0)	0 (0.0)	1 (0.0)	1162 (26.1)	202 (4.5)	4444 (100.0)
WOSCOPS	6595	54.71 (5.53)	6595 (100.0)	1690 (25.6)	6595 (100.0)	0 (0.0)	0 (0.0)	1039 (15.8)	76 (1.2)	499 (7.6)
CARE	4159	58.63 (9.33)	3583 (86.2)	932 (22.4)	4159 (100.0)	0 (0.0)	0 (0.0)	3375 (81.1)	586 (14.1)	4159 (100.0)
AFCAPS/TexCaps	6605	58.16 (7.25)	5608 (84.9)	818 (12.4)	5860 (88.7)	206 (3.1)	539 (8.2)	1448 (21.9)	155 (2.3)	19 (0.3)
LIPID	9014	60.80 (8.45)	7498 (83.2)	2408 (26.7)	0 (0.0)	0 (0.0)	9014 (100.0)	6822 (75.7)	782 (8.7)	9014 (100.0)
GISSI-Prevention	4271	59.37 (10.45)	3684 (86.3)	506 (11.8)	4271 (100.0)	0 (0.0)	0 (0.0)	959 (22.5)	582 (13.6)	4271 (100.0)
HPS	20536	63.46 (8.41)	15454 (75.3)	2913 (14.2)	19901 (96.9)	218 (1.1)	417 (2.0)	8457 (41.2)	5963 (29.0)	17375 (84.6)
ASCOT	10305	63.15 (8.53)	8363 (81.2)	3173 (30.8)	9752 (94.6)	271 (2.6)	282 (2.7)	8288 (80.4)	2527 (24.5)	1445 (14.0)
PROSPER	5804	74.83 (3.37)	2804 (48.3)	1558 (26.8)	5804 (100.0)	0 (0.0)	0 (0.0)	3592 (61.9)	623 (10.7)	2550 (43.9)
CARDS	2838	61.65 (8.12)	1929 (68.0)	649 (22.9)	2676 (94.3)	65 (2.3)	97 (3.4)	2377 (83.8)	2838 (100.0)	100 (3.5)
ALERT	2102	49.72 (10.94)	1387 (66.0)	389 (18.5)	2039 (97.0)	8 (0.4)	55 (2.6)	1575 (74.9)	396 (18.8)	400 (19.0)
ALLHAT	10355	66.62 (7.62)	5304 (51.2)	2401 (23.2)	5811 (56.1)	3911 (37.8)	633 (6.1)	9304 (89.9)	3638 (35.1)	2318 (22.4)
ALLIANCE	2442	61.20 (8.78)	2008 (82.2)	475 (19.5)	2048 (83.9)	278 (11.4)	116 (4.8)	0 (0.0)	540 (22.1)	2442 (100.0)
ASPEN	2410	60.52 (8.16)	1599 (66.3)	300 (12.4)	2029 (84.2)	155 (6.4)	226 (9.4)	1328 (55.1)	2410 (100.0)	747 (31.0)
MEGA	8214	58.26 (7.26)	2623 (31.9)	1257 (15.3)	0 (0.0)	0 (0.0)	8214 (100.0)	3188 (38.8)	1686 (20.5)	95 (1.2)
JUPITER	17802	66.13 (7.72)	11001 (61.8)	2820 (15.8)	12683 (71.2)	2224 (12.5)	2895 (16.3)	8036 (45.1)	76 (0.4)	0 (0.0)
**Total**	117896	62.43 (9.18)	83057 (70.4)	23427 (19.9)	88071 (74.7)	7336 (6.2)	22489 (19.1)	60950 (51.7)	23080 (19.6)	49878 (42.3)

SSSS, Scandinavian Simvastatin Survival Study; WOSCOPS, West of Scotland Coronary Prevention Study; CARE, Cholesterol And Recurrent Events; AFCAPS/TexCAPS, Air Force/Texas Coronary Atherosclerosis Prevention Study; LIPID, Long-term Intervention with Pravastatin in Ischaemic Disease; GISSI-Prevention, Gruppo Italiano per lo Studio della Sopravvivenza nell'Insuffi cienza cardiaca; HPS, Heart Protection Study; ASCOT, Anglo-Scandinavian Cardiac Outcomes Trial; PROSPER, PROspective Study of Pravastatin in the Elderly at Risk; CARDS, Collaborative Atorvastatin Diabetes Study; ALERT, Assessment of Lescol in Renal Transplantation; ALLHAT, Antihypertensive and Lipid-Lowering Treatment to Prevent Heart Attack Trial; ALLIANCE, Aggressive Lipid-Lowering Initiation Abates New Cardiac Events; ASPEN, Atorvastatin Study for Prevention of Coronary Heart Disease Endpoints in Non-Insulin-Dependent Diabetes Mellitus; MEGA, Management of Elevated Cholesterol in the Primary Prevention Group of Adult Japanese; JUPITER, Justification for the Use of Statins in Prevention: an Intervention Trial Evaluating Rosuvastatin.

Supplementary Table S3: Number of study participants with events during follow-up

	CTT Collaboration		UK Biobank	
	**Without CVD history**	**With CVD history**	**Without CVD history**	**With CVD history**
**Number participants**	**68,018**	**49,878**	**444,576**	**57,278**
MI	1758	4134	5427	2507
Stroke	1133	2160	4806	2132
Coronary revascularisation	1424	4958	6860	3451
Incident cancer	3085	3450	29682	5221
Incident diabetes*	NA	NA	9014	2772
Vascular death	1118	3751	2142	1708
Nonvascular death	1767	2242	11004	3170

*Not available in phase 1 of CTT database used in this study. CVD, cardiovascular disease; MI, myocardial infarction.

Supplementary Table S4: Parametric proportional hazards risk equations: myocardial infarction and stroke

	Myocardial infarction		Stroke	
Population	Without CVD history	With CVD history	Without CVD history	With CVD history
Parametric distribution	Weibull	Exponential	Weibull	Exponential
	Hazard ratio (95% CI)			
**Baseline variable**				
Male sex	2.4 (1.95-2.95)	1.55 (1.4-1.72)	1.09 (0.94-1.26)	1.01 (0.91-1.12)
Ethnicity (ref = white)				
Black ethnicity	0.55 (0.4-0.77)	0.72 (0.46-1.14)		
South Asian ethnicity	1.7 (1.43-2.03)	1.64 (1.31-2.04)		
Other ethnicity	0.93 (0.76-1.15)	0.92 (0.67-1.27)		
Townsend deprivation score quintiles (ref = 3rd quintile)				
Townsend score 1st quintile			0.91 (0.83-0.99)	0.8 (0.7-0.91)
Townsend score 2nd quintile			1.01 (0.92-1.12)	1.01 (0.87-1.16)
Townsend score 4th quintile			1.09 (0.98-1.2)	0.98 (0.85-1.14)
Townsend score 5th quintile			1.27 (1.15-1.41)	1.15 (1-1.33)
Smoking status (ref = non-smoker)				
Ex-smoker	1.14 (1.07-1.21)	1.22 (1.12-1.34)	1.08 (1.01-1.15)	1.03 (0.93-1.13)
Current smoker	2.28 (2.12-2.45)	2.15 (1.92-2.42)	1.86 (1.71-2.02)	1.53 (1.35-1.74)
Unhealthy diet	1.11 (1.05-1.17)		1.17 (1.1-1.24)	
Physical activity (ref = moderate)				
Physical activity: low	1.14 (1.05-1.23)			1.25 (1.11-1.42)
Physical activity: high	1.06 (1-1.14)			0.99 (0.88-1.12)
Physical activity: missing	1.14 (1.06-1.23)			1.16 (1.03-1.3)
LDL cholesterol (centred at 3.6) per 1 mmol/L	1.56 (1.51-1.61)	1.19 (1.12-1.26)		
Natural logarithm of HDL cholesterol (lnmmol/L)	0.32 (0.23-0.44)	0.58 (0.49-0.69)	0.71 (0.56-0.9)	
On treatment for hypertension	1.53 (1.26-1.87)	1.3 (1.18-1.42)	1.39 (1.2-1.61)	
Systolic Blood Pressure (centred at 140mmHg per 20mmHg)	1.27 (1.16-1.38)	1.06 (1.02-1.11)	1.31 (1.2-1.42)	1.2 (1.15-1.25)
Natural logarithm of creatinine (lnµmol/L)		2.15 (1.73-2.68)	1.66 (1.25-2.21)	1.52 (1.23-1.88)
BMI categories (ref = BMI 18.5-25 healthy)				
Underweight (BMI <18.5)			2.05 (1.32-3.18)	
Overweight (BMI 25-30)			0.87 (0.76-1.01)	
Obese I (BMI 30-35)			0.76 (0.63-0.92)	

Obese II (BMI 35-40)			0.83 (0.62-1.1)	
Obese III (BMI 40+)			0.72 (0.46-1.13)	
Severe mental illness	1.25 (1.13-1.37)		1.26 (1.14-1.39)	1.27 (1.12-1.43)
Type 1 diabetes at baseline	1.67 (1.31-2.12)	1.54 (1.29-1.84)	1.56 (1.23-1.98)	1.75 (1.43-2.15)
CVD history (ref = other CHD only)				
Previous MI only		1.51 (1.33-1.7)		1.03 (0.91-1.18)
History of a stroke only		0.85 (0.68-1.06)		2.01 (1.67-2.42)
History of peripheral artery disease only		0.89 (0.71-1.13)		1.33 (1.09-1.61)
2 or more disease histories		1.77 (1.53-2.05)		1.72 (1.48-1.98)
**Time-updating variables**				
Current age centred at 60 (per 10 years)	1.68 (1.61-1.75)	1.34 (1.26-1.43)	1.91 (1.74-2.09)	1.59 (1.48-1.71)
Incident MI (ref = none)				
Any incident MI			1.75 (1.3-2.38)	1.5 (1.26-1.78)
Incident stroke (ref = none)				
Incident stroke in same year		0.7 (0.43-1.15)		
Incident stroke in a previous year		1.61 (1.22-2.14)		
Incident coronary revascularisation (ref = none)				
Incident coronary revascularisation in same year		1.07 (0.8-1.42)		
Incident coronary revascularisation in a previous year		0.5 (0.38-0.65)		
Any incident coronary revascularisation	2.5 (1.51-4.14)		1.77 (1.26-2.47)	
Diabetes (ref = no diabetes, HbA1c 32-37)				
No diabetes, HbA1c<32 mmol/mol	0.9 (0.82-0.99)	0.81 (0.68-0.97)	1.03 (0.94-1.12)	1.02 (0.86-1.21)
No diabetes, HbA1c 37-42 mmol/mol	1.1 (1.03-1.18)	1.09 (0.98-1.21)	1.1 (1.02-1.18)	1.1 (0.98-1.23)
No diabetes, HbA1c 42-48 mmol/mol	1.29 (1.13-1.47)	1.26 (1.06-1.49)	1.28 (1.11-1.48)	1.12 (0.92-1.36)
Diabetes duration 0-10 years	1.29 (1.13-1.48)	1.26 (1.1-1.44)	1.25 (1.1-1.42)	1.47 (1.27-1.69)
Diabetes duration 10+ years	1.92 (1.61-2.3)	1.88 (1.64-2.16)	1.65 (1.43-1.9)	1.56 (1.32-1.83)
Cancer (ref = none)				
Incident cancer 0-5 years ago	1.22 (1.07-1.39)	1.33 (1.12-1.58)	1.85 (1.65-2.08)	1.66 (1.4-1.98)
Baseline cancer 0-5 years ago	1.28 (0.95-1.71)	0.83 (0.55-1.27)	1.4 (1.01-1.93)	1.44 (1-2.08)
All cancer 5+ years ago	1.02 (0.92-1.13)	1.13 (0.99-1.29)	1.35 (1.23-1.48)	1.24 (1.09-1.42)
**Interactions**				
Current age * systolic BP			0.89 (0.84-0.95)	
Current age * diabetes 0-10 years	0.93 (0.79-1.09)			
Current age * diabetes 10+ years	0.92 (0.77-1.1)			
**Shape***	1.04	NA	1.12	NA

*Shape > 1 for Weibull distribution and shape > 0 for Gompertz distribution indicate risk increases over time; otherwise decreases over time. CVD, cardiovascular disease; CHD, coronary heart disease; BP, blood pressure; BMI, body mass index; LDL, low-density lipoprotein; HDL, high-density lipoprotein; MI, myocardial infarction; HbA1c, hemoglobin A1c.

Supplementary Table S5: Parametric proportional hazards risk equations: coronary revascularisation, incident cancer and incident diabetes

	Coronary revascularisation		Cancer	Diabetes
Population	Without CVD history	With CVD history	Without cancer history	Without diabetes history
Parametric distribution	Weibull	Gompertz	Gompertz	Weibull
	Hazard ratio (95% CI)			
**Baseline variable**				
Male sex	2.18 (1.78-2.67)	2.21 (2.02-2.42)	1.03 (1-1.05)	1.32 (1.23-1.41)
Ethnicity (ref = white)				
Black ethnicity	0.55 (0.39-0.76)	0.63 (0.39-1)	0.96 (0.86-1.06)	1.07 (0.88-1.29)
South Asian ethnicity	1.57 (1.36-1.8)	1.41 (1.18-1.68)	0.73 (0.65-0.81)	1.24 (1.06-1.44)
Other ethnicity	0.91 (0.76-1.1)	1.29 (1.01-1.64)	0.85 (0.78-0.93)	1.49 (1.3-1.72)
Townsend deprivation score quintiles (ref = 3rd quintile)				
Townsend score 1st quintile				0.91 (0.85-0.99)
Townsend score 2nd quintile				0.98 (0.9-1.06)
Townsend score 4th quintile				1.03 (0.95-1.12)
Townsend score 5th quintile				1.12 (1.03-1.22)
Smoking status (ref = non-smoker)				
Ex-smoker	1.15 (1.09-1.21)	1.16 (1.07-1.25)	1.14 (1.11-1.17)	1.07 (1.01-1.13)
Current smoker	1.17 (1.09-1.25)	1.3 (1.17-1.45)	1.44 (1.39-1.5)	0.96 (0.89-1.04)
Unhealthy diet			1.05 (1.03-1.08)	1.13 (1.07-1.19)
Physical activity (ref = moderate)				
Physical activity: low	0.9 (0.83-0.96)		1.07 (1.04-1.1)	1.07 (1-1.15)
Physical activity: high	1.07 (1-1.13)		0.98 (0.95-1.01)	0.89 (0.84-0.96)
Physical activity: missing	1.03 (0.97-1.11)		1.03 (1-1.06)	1.07 (1-1.14)
LDL cholesterol (centred at 3.6) per 1 mmol/L	1.54 (1.35-1.76)	1.22 (1.14-1.31)		
Natural logarithm of HDL cholesterol (lnmmol/L)	0.24 (0.17-0.33)	0.41 (0.33-0.5)	0.93 (0.88-0.99)	0.3 (0.26-0.34)
On treatment for hypertension	1.61 (1.33-1.95)	1.76 (1.56-1.97)		1.38 (1.3-1.46)
Systolic Blood Pressure (centred at 140mmHg per 20mmHg)				1.13 (1.1-1.16)
Diastolic Blood Pressure (centred at 80mmHg per 10mmHg)		0.89 (0.85-0.93)		
Natural logarithm of creatinine (lnµmol/L)				0.64 (0.55-0.74)
HbA1c (mmol/mol)				1.3 (1.29-1.31)
BMI categories (ref = BMI 18.5-25 healthy)				

Underweight (BMI <18.5)		0.39 (0.13-1.18)		1.38 (0.79-2.38)
Overweight (BMI 25-30)		1.11 (0.99-1.24)		1.51 (1.38-1.65)
Obese I (BMI 30-35)		1.02 (0.88-1.19)		2.39 (2.18-2.62)
Obese II (BMI 35-40)		0.93 (0.71-1.21)		3.38 (3.04-3.76)
Obese III (BMI 40+)		0.5 (0.3-0.84)		3.69 (3.24-4.19)
Severe mental illness				1.38 (1.29-1.48)
Type 1 diabetes at baseline			1.38 (1.24-1.53)	
CVD history (ref = other CHD only for the coronary revascularisation equation/none for the cancer and diabetes equations)				
Other CHD only			1 (0.94-1.06)	1.2 (1.11-1.31)
Previous MI only		0.74 (0.65-0.85)	0.98 (0.93-1.04)	1.36 (1.04-1.77)
History of a stroke only		0.18 (0.12-0.26)	0.99 (0.92-1.06)	1.41 (1.18-1.68)
History of peripheral artery disease only		0.15 (0.1-0.23)	1.21 (1.11-1.32)	1.31 (1.1-1.56)
2 or more disease histories		0.8 (0.68-0.95)	1.09 (1.02-1.15)	1.46 (1.32-1.61)
**Time-updating variables**				
Current age centred at 60 (per 10 years)	1.18 (1.06-1.3)	1.17 (1.1-1.24)	1.57 (1.53-1.61)	1.58 (1.16-2.15)
Incident MI (ref = none)				
Incident MI in same year	682.04 (565.35-822.8)	82.48 (70.57-96.39)	0.71 (0.58-0.86)	
Incident MI in previous year	75.99 (50.47-114.41)	31.31 (24.69-39.7)		
Incident MI at least 2 years ago	15.14 (8.7-26.34)	5.79 (4.25-7.89)		
Incident MI in a previous year			0.95 (0.86-1.04)	
Any incident MI				1.4 (1.12-1.74)
Incident stroke (ref = none)				
Incident stroke in same year		0.14 (0.05-0.35)		
Incident stroke in a previous year		0.67 (0.42-1.05)		
Incident coronary revascularisation (ref = none)				
Incident coronary revascularisation in same year			0.75 (0.63-0.89)	0.38 (0.21-0.68)
Incident coronary revascularisation in a previous year			0.97 (0.9-1.05)	0.99 (0.81-1.21)
Diabetes (ref = no diabetes, HbA1c 32-37)				
No diabetes, HbA1c<32 mmol/mol	0.94 (0.87-1.02)	0.88 (0.76-1.02)	1.03 (1-1.06)	
No diabetes, HbA1c 37-42 mmol/mol	1.1 (1.04-1.17)	1.11 (1.01-1.21)	1.04 (1.02-1.07)	
No diabetes, HbA1c 42-48 mmol/mol	1.16 (1.03-1.31)	1.22 (1.05-1.41)	1.16 (1.1-1.23)	
Diabetes duration 0-10 years	1.19 (1.09-1.31)	1.26 (1.13-1.4)	1.12 (1.06-1.17)	
Diabetes duration 10+ years	1.53 (1.38-1.7)	1.32 (1.18-1.47)	1.07 (1.01-1.14)	
Cancer (ref = none)				

Incident cancer 0-5 years ago	0.73 (0.64-0.84)	0.64 (0.52-0.78)		2.45 (2.22-2.69)
Baseline cancer 0-5 years ago	1.09 (0.82-1.44)	0.85 (0.61-1.2)		1.62 (1.23-2.14)
All cancer 5+ years ago	1.06 (0.97-1.16)	0.89 (0.79-1)		1.34 (1.24-1.46)
**Interactions**				
Current age * male			1.52 (1.48-1.57)	
Current age * current smoker			1.13 (1.08-1.17)	
Current age * ex-smoker			1 (0.97-1.03)	
Current age * HbA1c				0.99 (0.98-1)
**Shape***	1.13	-0.04	-0.01	1.44

*Shape > 1 for Weibull distribution and shape > 0 for Gompertz distribution indicate risk increases over time; otherwise decreases over time. CVD, cardiovascular disease; CHD, coronary heart disease; BP, blood pressure; BMI, body mass index; LDL, low-density lipoprotein; HDL, high-density lipoprotein; MI, myocardial infarction; HbA1c, hemoglobin A1c.

Supplementary Table S6: Parametric proportional hazards risk equations: vascular death and nonvascular death

	**Vascular death**		**Nonvascular death**	
Population	Without CVD history	With CVD history	Without CVD history	With CVD history
Parametric distribution	Gompertz	Gompertz	Gompertz	Gompertz
	**Hazard ratio (95% CI)**			
**Baseline variables**				
Male sex	1.53 (1.21-1.93)	1.33 (1.18-1.49)	1.36 (1.3-1.43)	1.01 (0.89-1.14)
Ethnicity (ref = white)				
Black ethnicity			0.68 (0.56-0.83)	0.62 (0.42-0.92)
South Asian ethnicity			0.77 (0.63-0.96)	0.71 (0.5-1.01)
Other ethnicity			0.83 (0.7-0.97)	0.8 (0.57-1.12)
Townsend deprivation score quintiles (ref = 3rd quintile)				
Townsend score 1st quintile	0.68 (0.6-0.78)	0.76 (0.65-0.88)	0.93 (0.88-0.98)	0.82 (0.73-0.92)
Townsend score 2nd quintile	0.76 (0.66-0.88)	0.91 (0.77-1.08)	0.93 (0.87-0.99)	0.99 (0.88-1.12)
Townsend score 4th quintile	1.09 (0.94-1.26)	1.11 (0.94-1.3)	1.04 (0.97-1.11)	1 (0.89-1.14)
Townsend score 5th quintile	1.2 (1.04-1.38)	1.14 (0.98-1.33)	1.27 (1.19-1.36)	1.34 (1.19-1.5)
Smoking status (ref = non-smoker)				
Ex-smoker	1.03 (0.93-1.13)	1.33 (1.18-1.49)	1.21 (1.16-1.27)	1.31 (1.2-1.42)
Current smoker	2.16 (1.93-2.43)	1.97 (1.71-2.28)	1.97 (1.86-2.08)	1.9 (1.71-2.11)
Unhealthy diet	1.19 (1.09-1.3)		1.15 (1.11-1.2)	1.26 (1.18-1.36)
Physical activity (ref = moderate)				
Physical activity: low		1.06 (0.92-1.21)	1.24 (1.17-1.31)	1.33 (1.2-1.46)
Physical activity: high		0.95 (0.83-1.09)	0.96 (0.91-1)	0.93 (0.85-1.03)
Physical activity: missing		1.25 (1.09-1.42)	1.11 (1.05-1.16)	1.12 (1.02-1.24)
LDL cholesterol (centred at 3.6) per 1 mmol/L	1.28 (1.11-1.49)	1.17 (1.07-1.27)	0.88 (0.82-0.95)	
Natural logarithm of HDL cholesterol (lnmmol/L)		0.83 (0.7-0.97)		
On treatment for hypertension	1.45 (1.14-1.83)	1.3 (1.19-1.43)		
Systolic Blood Pressure (centred at 140mmHg per 20mmHg)				
Diastolic Blood Pressure (centred at 80mmHg per 10mmHg)		0.94 (0.9-0.97)		
Natural logarithm of creatinine (lnµmol/L)	2.19 (1.44-3.34)	3.98 (3.25-4.87)		1.85 (1.47-2.33)
BMI categories (ref = BMI 18.5-25 healthy)				
Underweight (BMI <18.5)		1.78 (1.14-2.79)	2.15 (1.63-2.85)	2.81 (1.96-4.03)
Overweight (BMI 25-30)		0.88 (0.8-0.97)	0.88 (0.79-0.97)	0.87 (0.78-0.96)
Obese I (BMI 30-35)		0.96 (0.85-1.09)	0.74 (0.65-0.85)	0.83 (0.72-0.96)

Obese II (BMI 35-40)		1.12 (0.89-1.4)	0.81 (0.65-1)	0.94 (0.73-1.21)
Obese III (BMI 40+)		1.73 (1.22-2.44)	0.94 (0.71-1.25)	1.33 (0.89-1.98)
Severe mental illness	1.34 (1.17-1.55)		1.14 (1.07-1.22)	
Type 1 diabetes at baseline			1.19 (1.01-1.41)	
CVD history (ref = other CHD only)				
Previous MI only		1.54 (1.35-1.75)		1.14 (0.99-1.31)
History of a stroke only		1.32 (1.07-1.63)		1.54 (1.27-1.88)
History of peripheral artery disease only		1.38 (1.12-1.7)		1.58 (1.3-1.91)
2 or more disease histories		1.85 (1.6-2.13)		1.39 (1.2-1.61)
**Time-updating variables**				
Current age centred at 60 (per 10 years)	1.93 (1.79-2.07)	1.6 (1.43-1.8)	1.72 (1.65-1.79)	1.55 (1.43-1.68)
Incident MI (ref = none)				
Any incident MI				1.62 (1.35-1.93)
Incident MI in same year	155.63 (136.92-176.91)	34.37 (30.05-39.32)		
Incident MI in previous year	8.47 (5.93-12.1)	7.48 (6.07-9.22)		
Incident MI at least 2 years ago	2.9 (2.08-4.04)	4.92 (4.05-5.96)		
Incident stroke (ref = none)				
Incident stroke in same year	64.26 (56.28-73.37)	13.6 (11.7-15.8)		
Incident stroke in a previous year	8.25 (6.76-10.07)			
Incident stroke in previous year		4.13 (3.15-5.41)		
Incident stroke at least 2 years ago		1.99 (1.51-2.61)		
Any incident stroke			1.97 (1.54-2.52)	1.97 (1.64-2.37)
Incident coronary revascularisation (ref = none)				
Incident coronary revascularisation in same year	0.26 (0.15-0.45)			0.45 (0.28-0.74)
Incident coronary revascularisation in a previous year	0.84 (0.44-1.61)			0.98 (0.8-1.21)
Any incident coronary revascularisation		0.5 (0.43-0.59)		
Diabetes (ref = no diabetes, HbA1c 32-37)				
No diabetes, HbA1c<32 mmol/mol	1.11 (0.96-1.29)	1.08 (0.86-1.34)	1.04 (0.98-1.1)	1.15 (1-1.33)
No diabetes, HbA1c 37-42 mmol/mol	1.31 (1.18-1.46)	1.07 (0.93-1.22)	1.1 (1.05-1.16)	1.12 (1.02-1.23)
No diabetes, HbA1c 42-48 mmol/mol	1.79 (1.46-2.19)	1.28 (1.04-1.58)	1.32 (1.19-1.46)	1.08 (0.91-1.27)
Diabetes duration 0-10 years	2.61 (2.12-3.22)	1.94 (1.57-2.39)	1.89 (1.76-2.02)	1.74 (1.56-1.95)
Diabetes duration 10+ years	4.5 (3.6-5.64)	2.17 (1.75-2.7)	1.45 (1.31-1.59)	1.76 (1.57-1.98)
Cancer (ref = none)				
Incident cancer 0-5 years ago		1.12 (0.92-1.36)		
Baseline cancer 0-5 years ago		1.78 (1.17-2.7)		

All cancer 5+ years ago		0.85 (0.72-1)		
Incident cancer in same year			84.4 (79.39-89.73)	38.89 (35.01-43.21)
Incident cancer 1 year ago			98.29 (92.38-104.59)	39.66 (35.41-44.43)
Incident cancer 2 years ago			60.54 (56.08-65.36)	19.97 (17-23.45)
Incident cancer 3 years ago			38.8 (35.19-42.79)	14.43 (11.8-17.66)
Incident cancer 4 years ago			30.39 (26.97-34.24)	11.41 (8.89-14.65)
Baseline cancer 1 year ago			21.46 (14.45-31.89)	29.36 (19.31-44.64)
Baseline cancer 2 years ago			33.43 (27.07-41.29)	18.33 (12.95-25.93)
Baseline cancer 3 years ago			33.72 (28.47-39.95)	13.44 (9.68-18.66)
Baseline cancer 4 year ago			24.13 (20.34-28.62)	11.52 (8.46-15.68)
All cancer 5-10 years ago			16.52 (15.37-17.76)	8.13 (7.18-9.22)
All cancer 10-15 years ago			13.02 (11.91-14.23)	6.32 (5.38-7.43)
All cancer 15-20 years ago			10.82 (9.62-12.17)	5.63 (4.54-7)
All cancer 20+ years ago			8.29 (7.46-9.22)	4.67 (3.88-5.62)
**Interactions**				
Current age * LDL cholesterol		0.87 (0.8-0.96)		
Current age * male			0.82 (0.78-0.87)	
Current age * diabetes 0-10 years	0.75 (0.61-0.93)	0.74 (0.59-0.91)		
Current age * diabetes 10+ years	0.69 (0.55-0.87)	0.68 (0.55-0.84)		
MI same year * diabetes 0-10 years	0.92 (0.65-1.3)			
MI same year * diabetes 10+ years	0.5 (0.34-0.74)			
Stroke same year * diabetes 0-10 years	0.42 (0.27-0.66)			
Stroke same year * diabetes 10+ years	0.2 (0.12-0.33)			
**Shape***	0.05	0.07	0.06	0.05

*Shape > 1 for Weibull distribution and shape > 0 for Gompertz distribution indicate risk increases over time; otherwise decreases over time. CVD, cardiovascular disease; CHD, coronary heart disease; BP, blood pressure; BMI, body mass index; CRV, coronary revascularisation; LDL, low-density lipoprotein; HDL, high-density lipoprotein; MI, myocardial infarction; HbA1c, hemoglobin A1c.

Supplementary Table S7: The health-related quality of life (QoL) model

MODEL	Model on pooled 2006, 2011, 2017 HSE data<br>N = 24,231 participants<br>Coefficient (95% CI)
Intercept	0.88 (0.87 , 0.89)
Age spline 1 (<70)*	-0.03 (-0.03 , -0.02)
Age spline 2 (>=70)*	-0.06 (-0.07 , -0.05)
Male	0.03 (0.03 , 0.04)
Ethnicity (ref = white)	
Black	0 (-0.02 , 0.02)
South Asian	-0.03 (-0.04 , -0.01)
Others	-0.02 (-0.04 , 0)
Deprivation quintiles (ref = IMD 3)	
IMD 1	0.03 (0.02 , 0.03)
IMD 2	0.01 (0 , 0.02)
IMD 4	-0.02 (-0.03 , -0.01)
IMD 5	-0.06 (-0.06 , -0.05)
BMI categories (ref = 18.5-25)	
BMI <18.5	-0.03 (-0.06 , 0)
BMI 25-30	-0.01 (-0.02 , -0.01)
BMI 30-35	-0.04 (-0.05 , -0.03)
BMI 35-40	-0.08 (-0.1 , -0.07)
BMI 40+	-0.12 (-0.15 , -0.1)
Current smoker	-0.06 (-0.07 , -0.05)
Ex-smoker	-0.02 (-0.03 , -0.01)
Treatment of hypertension	-0.02 (-0.03 , -0.02)
Mental illness	-0.26 (-0.27 , -0.24)
Cancer†	-0.13 (-0.14 , -0.11)
Diabetes <=10 years	-0.04 (-0.06 , -0.03)
Diabetes >10 years	-0.08 (-0.1 , -0.06)
CVD history	
Angina ever only	-0.12 (-0.14 , -0.1)
MI 1year plus only	-0.07 (-0.1 , -0.04)
Stroke 1year plus only	-0.13 (-0.16 , -0.11)
Two or more	-0.19 (-0.21 , -0.16)
Coronary revascularisation 1 year plus	0.04 (0.02 , 0.07)
MI in 12 months	-0.1 (-0.16 , -0.03)
Stroke in 12 months	-0.09 (-0.13 , -0.04)
Coronary revascularisation in 12 months	0.02 (-0.06 , 0.1)

*Impact of age on QoL is predicted as follows: when age < 70, QoL=Age spline 1 * (age - 60)/10; when age >= 70, QoL=Age spline 1 + Age spline 2 * [(age - 60)/10 – 1]. †the cancer-related decrement in QoL has been modified to 0.03 informed from literature(2-8) in recognition that the HSE recorded only cancer affecting daily life, whereas cancer in the model includes any cancer, except non-melanoma skin cancer. HSE, Health Survey for England; IMD, index of Multiple Deprivation; BMI, body mass index; CVD, cardiovascular disease; MI, myocardial infarction.

Supplementary Table S8: Lifelong simulation results (95% CI), men without CVD history

Age at entry	10-year CVD risk	Life years	QALYs	MI	Stroke	Coronary revascularisation	Diabetes*	Cancer*	Vascular death	Nonvascular death
				Number of individuals experiencing at least one event per 1000 individuals						
40-49	<5%	38.9 (37.3 to 40.2)	33 (31.8 to 34)	104 (91 to 116)	129 (106 to 149)	135 (121 to 147)	130 (118 to 143)	592 (518 to 654)	260 (168 to 376)	737 (623 to 826)
	5%-10%	35.4 (34 to 36.6)	28.5 (27.5 to 29.4)	167 (149 to 185)	146 (122 to 167)	206 (188 to 223)	195 (179 to 209)	581 (512 to 640)	306 (218 to 411)	692 (589 to 778)
	10%-15%	32.6 (31.2 to 33.7)	24.8 (23.9 to 25.5)	235 (213 to 257)	165 (138 to 189)	278 (256 to 297)	262 (243 to 278)	543 (476 to 601)	360 (275 to 459)	639 (541 to 722)
	15%-20%	30.7 (29.3 to 31.8)	22.3 (21.4 to 23)	287 (263 to 314)	182 (151 to 209)	330 (306 to 352)	316 (295 to 334)	505 (441 to 565)	405 (318 to 502)	594 (498 to 681)
	≥20%	28.5 (27.1 to 29.7)	19.4 (18.4 to 20.2)	356 (323 to 386)	207 (171 to 241)	399 (369 to 424)	367 (344 to 388)	447 (383 to 507)	473 (380 to 563)	527 (437 to 620)
50-59	<5%	34.6 (33.3 to 35.7)	29.4 (28.5 to 30.3)	79 (69 to 88)	137 (116 to 155)	96 (87 to 104)	96 (86 to 106)	671 (611 to 718)	219 (145 to 315)	772 (682 to 842)
	5%-10%	32.1 (31 to 33.1)	26.4 (25.6 to 27.2)	124 (111 to 137)	149 (128 to 168)	150 (137 to 161)	134 (122 to 146)	677 (621 to 722)	246 (175 to 336)	746 (660 to 813)
	10%-15%	29.8 (28.8 to 30.7)	23.6 (22.9 to 24.3)	176 (159 to 192)	164 (142 to 183)	207 (191 to 221)	181 (167 to 195)	670 (619 to 713)	279 (210 to 364)	715 (634 to 782)
	15%-20%	28 (27 to 28.8)	21.4 (20.7 to 21.9)	227 (207 to 246)	180 (156 to 201)	261 (243 to 277)	224 (208 to 239)	649 (599 to 692)	315 (250 to 396)	681 (603 to 746)
	≥20%	25.1 (24.2 to 25.9)	17.9 (17.3 to 18.5)	306 (282 to 333)	211 (184 to 237)	341 (320 to 361)	279 (261 to 295)	596 (547 to 641)	376 (304 to 451)	622 (549 to 692)
60-70	<5%	NaN (NA to NA)	NaN (NA to NA)	NaN (NA to NA)	NaN (NA to NA)	NaN (NA to NA)	NaN (NA to NA)	NaN (NA to NA)	NaN (NA to NA)	NaN (NA to NA)
	5%-10%	28.2 (27.3 to 29)	23.2 (22.5 to 23.8)	87 (78 to 97)	158 (137 to 176)	99 (91 to 108)	92 (82 to 101)	741 (700 to 775)	204 (148 to 275)	778 (711 to 828)
	10%-15%	26.6 (25.8 to 27.4)	21.3 (20.7 to 21.9)	132 (119 to 145)	169 (147 to 187)	150 (138 to 162)	124 (113 to 135)	738 (700 to 770)	233 (177 to 304)	752 (685 to 803)
	15%-20%	24.9 (24.2 to 25.6)	19.4 (18.9 to 19.9)	171 (156 to 186)	182 (160 to 201)	193 (179 to 207)	154 (142 to 166)	734 (697 to 765)	259 (202 to 326)	728 (665 to 781)
	≥20%	22.3 (21.6 to 22.9)	16.5 (16 to 16.9)	249 (228 to 270)	210 (187 to 231)	274 (256 to 292)	199 (187 to 214)	708 (672 to 738)	308 (255 to 371)	683 (623 to 735)

CVD, cardiovascular disease; QALY, quality-adjusted life year; MI, myocardial infarction; NA, no such participants in the UK Biobank cohort. * Rates for incident diabetes and incident cancer are based on individuals without baseline diabetes or baseline cancer, respectively

Supplementary Table S9: Lifelong simulation results (95% CI), women without CVD history

Age at entry	10-year CVD risk	Life years	QALYs	MI	Stroke	Coronary revascularisation	Diabetes*	Cancer*	Vascular death	Nonvascular death
				Number of individuals experiencing at least one event per 1000 individuals						
40-49	<5%	42.1 (40.3 to 43.7)	33 (31.8 to 34)	42 (36 to 48)	132 (108 to 153)	46 (41 to 51)	101 (90 to 111)	400 (358 to 442)	213 (119 to 343)	766 (645 to 858)
	5%-10%	36.7 (35.1 to 38)	24.6 (23.7 to 25.4)	102 (89 to 116)	161 (134 to 184)	110 (97 to 122)	245 (226 to 264)	412 (374 to 452)	293 (196 to 414)	699 (583 to 793)
	10%-15%	34.9 (33.2 to 36.2)	21.4 (20.5 to 22.2)	134 (117 to 155)	183 (152 to 212)	148 (130 to 164)	313 (292 to 334)	398 (358 to 438)	336 (232 to 455)	660 (545 to 760)
	15%-20%	34.8 (32.9 to 36.4)	21.1 (20 to 22)	163 (140 to 192)	196 (159 to 230)	186 (164 to 208)	372 (346 to 395)	380 (339 to 422)	361 (247 to 481)	635 (517 to 745)
	≥20%	32.7 (30.9 to 34.3)	19.3 (18.2 to 20.3)	181 (153 to 210)	215 (175 to 253)	192 (168 to 216)	485 (462 to 504)	400 (356 to 444)	364 (257 to 479)	634 (521 to 741)
50-59	<5%	37.2 (35.8 to 38.4)	29 (28 to 29.8)	49 (42 to 56)	149 (128 to 168)	49 (44 to 54)	97 (87 to 107)	422 (386 to 458)	212 (130 to 319)	729 (640 to 808)
	5%-10%	34.2 (32.9 to 35.3)	24.9 (24 to 25.5)	86 (75 to 97)	166 (142 to 184)	88 (79 to 97)	167 (153 to 181)	432 (398 to 467)	257 (170 to 360)	692 (609 to 770)
	10%-15%	31.4 (30.2 to 32.4)	20.8 (20.1 to 21.5)	132 (117 to 148)	189 (162 to 211)	137 (123 to 152)	249 (231 to 266)	434 (401 to 466)	306 (222 to 404)	664 (581 to 741)
	15%-20%	29.7 (28.4 to 30.7)	18.4 (17.7 to 19)	169 (148 to 196)	212 (181 to 239)	180 (162 to 202)	306 (286 to 325)	425 (392 to 458)	344 (256 to 438)	638 (551 to 718)
	≥20%	28 (26.6 to 29.1)	16.4 (15.6 to 17)	204 (175 to 240)	243 (206 to 274)	214 (190 to 243)	353 (332 to 375)	425 (389 to 461)	359 (264 to 457)	630 (539 to 717)
60-70	<5%	33.4 (32.3 to 34.3)	26.1 (25.3 to 26.8)	40 (35 to 46)	161 (141 to 179)	37 (33 to 41)	66 (59 to 75)	427 (395 to 459)	187 (119 to 273)	702 (634 to 766)
	5%-10%	31.5 (30.5 to 32.4)	23.6 (22.9 to 24.2)	68 (60 to 78)	170 (149 to 187)	66 (59 to 72)	112 (101 to 123)	433 (403 to 462)	220 (150 to 308)	674 (608 to 735)
	10%-15%	29.3 (28.3 to 30)	21 (20.4 to 21.5)	101 (89 to 114)	184 (162 to 201)	99 (90 to 108)	155 (142 to 168)	434 (406 to 462)	253 (184 to 337)	648 (584 to 705)
	15%-20%	27.5 (26.6 to 28.2)	18.8 (18.3 to 19.4)	136 (122 to 152)	200 (177 to 219)	136 (124 to 149)	197 (182 to 212)	436 (409 to 463)	286 (216 to 365)	630 (569 to 688)
	≥20%	25.2 (24.2 to 25.9)	16 (15.4 to 16.4)	186 (162 to 215)	234 (207 to 258)	190 (171 to 213)	240 (223 to 256)	437 (410 to 463)	323 (247 to 403)	622 (558 to 681)

CVD, cardiovascular disease; QALY, quality-adjusted life year; MI, myocardial infarction; NA, no such participants in the UK Biobank cohort. * Rates for incident diabetes and incident cancer are based on individuals without baseline diabetes or baseline cancer, respectively

Supplementary Table S10: Lifelong simulation results (95% CI), men and women with CVD history

Sex	Age at entry	Life years	QALYs	MI	Stroke	Coronary revascularisation	Diabetes*	Cancer*	Vascular death	Nonvascular death
				Number of individuals experiencing at least one event per 1000 individuals						
Men	40-49	30 (28.3 to 31.9)	20.8 (19.7 to 22)	183 (164 to 204)	130 (114 to 149)	178 (153 to 208)	221 (201 to 241)	398 (335 to 462)	421 (293 to 552)	579 (448 to 706)
	50-59	24.2 (23.1 to 25.5)	15.7 (15.1 to 16.5)	219 (202 to 240)	156 (140 to 174)	235 (212 to 262)	242 (224 to 263)	499 (446 to 548)	366 (276 to 469)	633 (530 to 723)
	60-70	19.5 (18.7 to 20.4)	12.2 (11.7 to 12.8)	232 (214 to 254)	175 (159 to 194)	265 (247 to 288)	233 (217 to 254)	596 (556 to 633)	317 (250 to 397)	680 (600 to 746)
Women	40-49	34.9 (32.6 to 37.3)	23.4 (22 to 24.7)	87 (76 to 101)	135 (116 to 160)	49 (40 to 61)	141 (126 to 158)	336 (297 to 380)	364 (217 to 517)	634 (481 to 777)
	50-59	29.8 (28 to 31.6)	18.2 (17.3 to 19.2)	121 (106 to 139)	175 (154 to 200)	80 (68 to 95)	194 (177 to 215)	371 (339 to 407)	324 (213 to 456)	662 (530 to 773)
	60-70	25.4 (24.1 to 26.7)	14.7 (14 to 15.5)	147 (128 to 170)	210 (188 to 235)	112 (98 to 131)	215 (196 to 235)	393 (366 to 421)	294 (205 to 405)	661 (556 to 748)

CVD, cardiovascular disease; QALY, quality-adjusted life year; MI, myocardial infarction.

* Rates for incident diabetes and incident cancer are based on individuals without baseline diabetes or baseline cancer, respectively

Supplementary Figure S1: Development and structure of the CVD microsimulation model

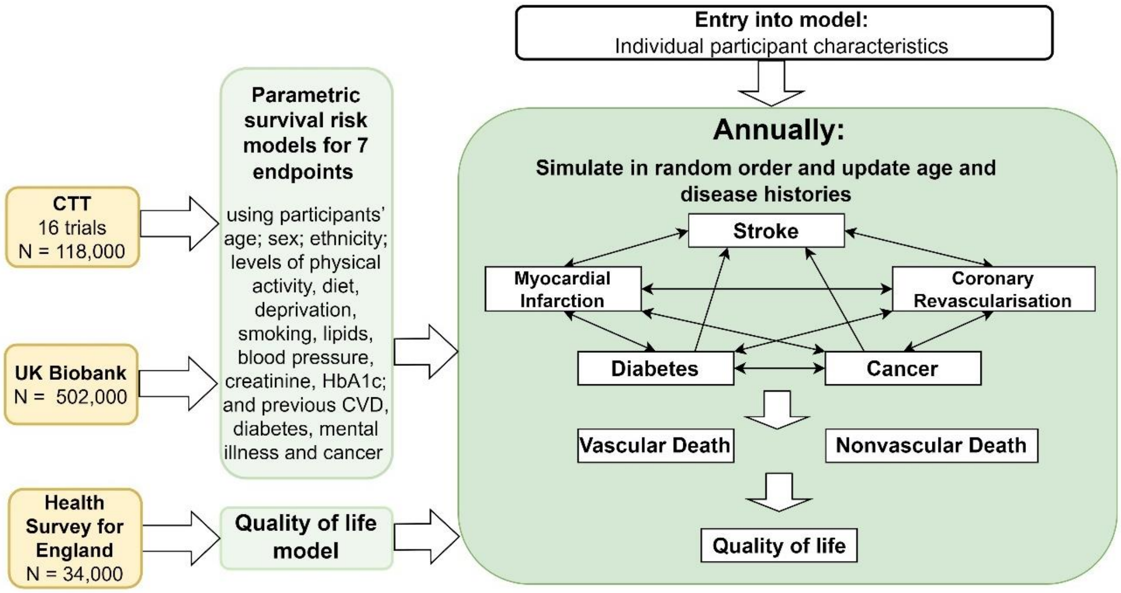

The model inputs are in the form of individual patient characteristics. The risks of first post entry occurrences of events of each type are simulated annually in a random order until pre-specified age or death. During annual cycles, an individual can experience one or more key events or die from vascular or nonvascular cause. The experience of events influences the future event risks in a time-dependent manner, with histories of events and age updated annually. The arrows indicate the direction of associations between any two endpoints. Patient's quality of life is calculated annually informed by patient characteristics and experienced events. CTT, Cholesterol Treatment Trialists' Collaboration; BMI, body mass index; CVD, cardiovascular disease; HbA1c, Hemoglobin A1C..

Supplementary Figure S2: Internal validation of the first-stage CVD model based on CTT data

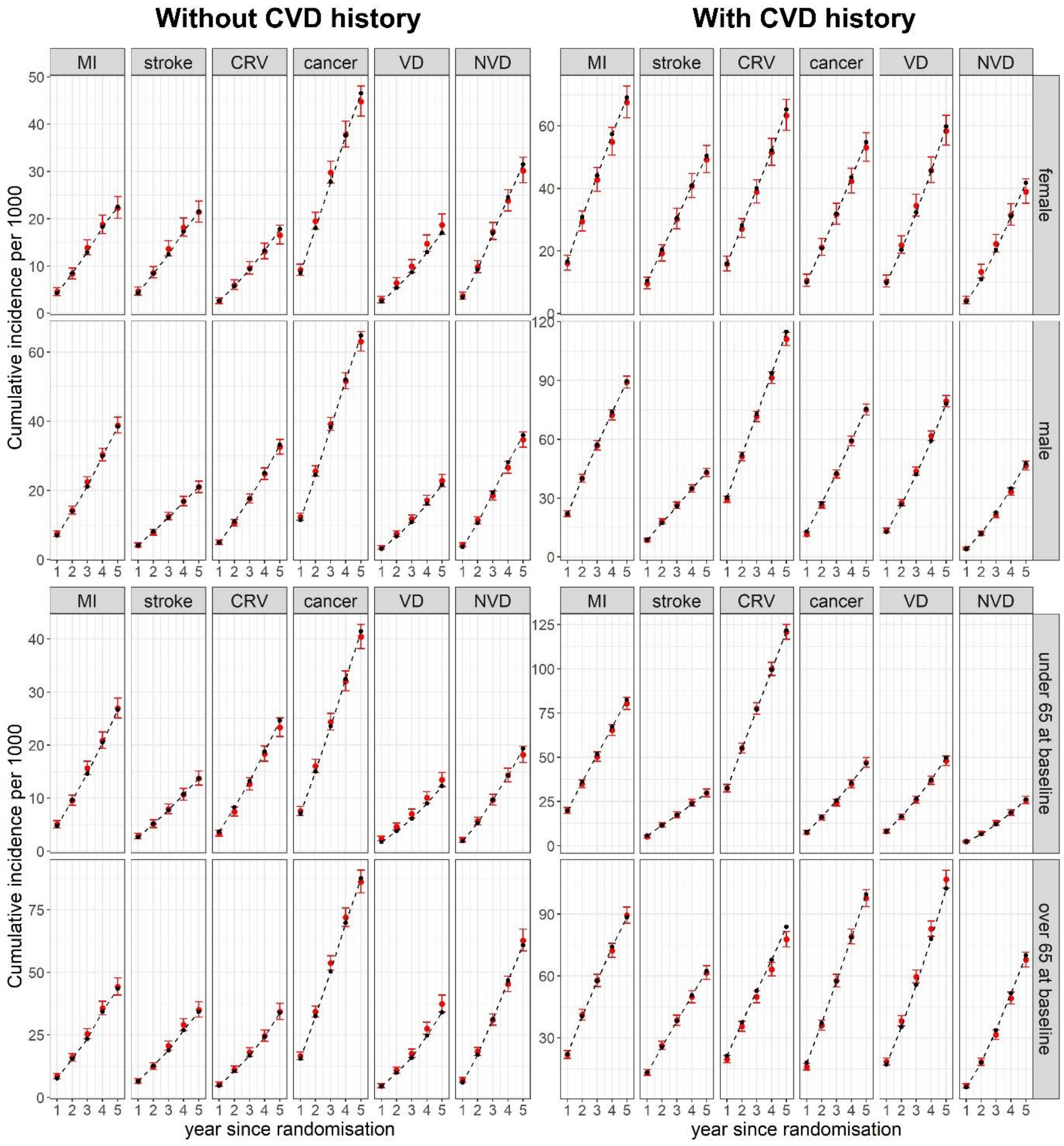

RED=Observed; BLACK=model. CVD, cardiovascular disease; MI, myocardial infarction; CRV, coronary revascularisation; VD, vascular death; NVD, non-vascular death.

Supplementary Figure S3: Validation of the calibrated CVD microsimulation model in the UKB cohort by sex, age, ethnicity and history of diabetes at entry

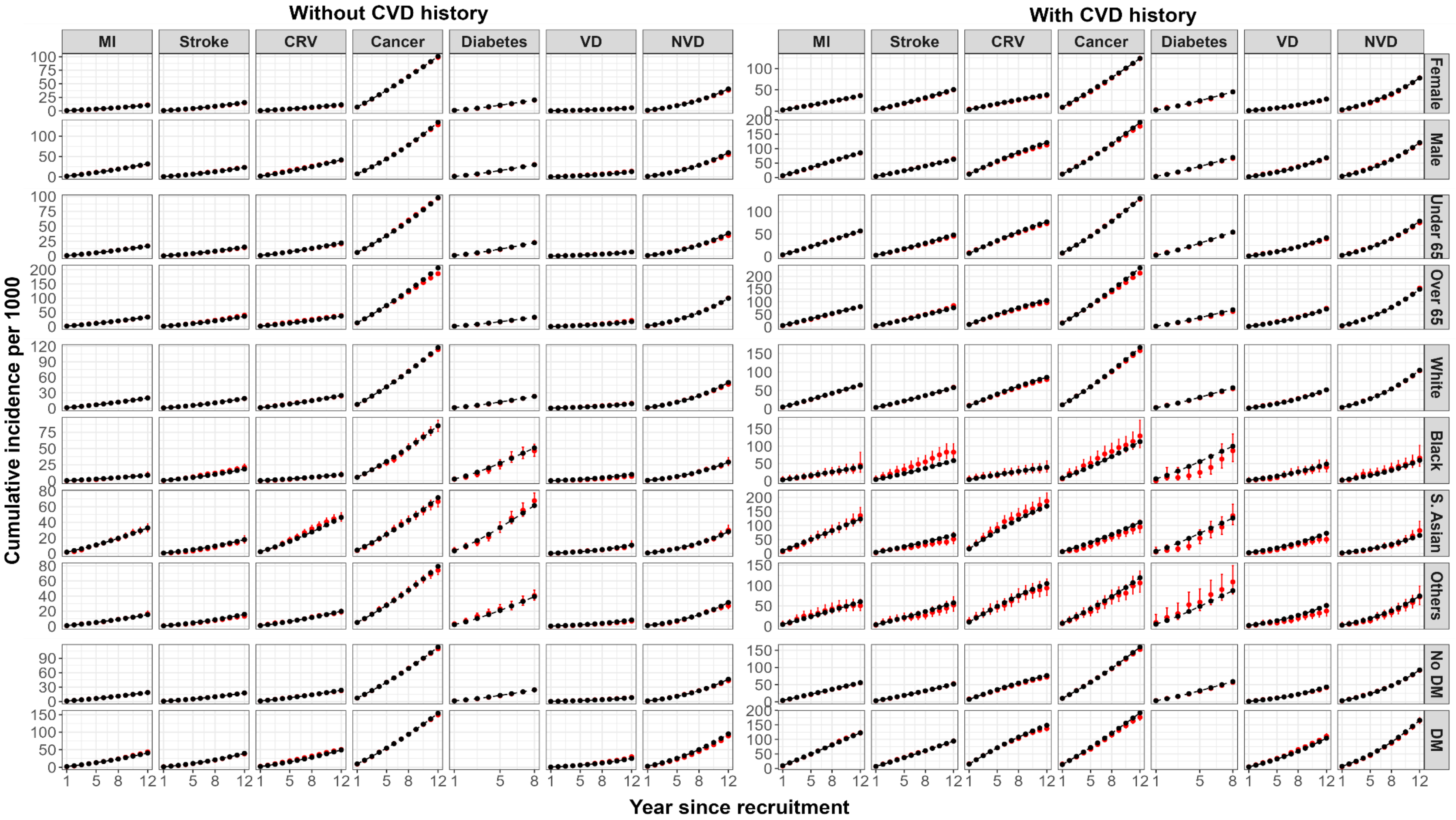

RED=Observed; BLACK=model. Validation includes three extra years that were not used to calibrate the model. MI, myocardial infarction; CRV, coronary revascularisation; VD, vascular death; NVD, non-vascular death; S. Asian, South Asian; DM, (baseline) diabetes mellitus. Follow-up of incident diabetes partly relies on primary care records, which ended earlier than other data types in the UKB. Incident diabetes is not predicted for individuals with diabetes history.

Supplementary Figure S4: Validation of the calibrated CVD microsimulation model in the UKB cohort by quintile of socioeconomic deprivation

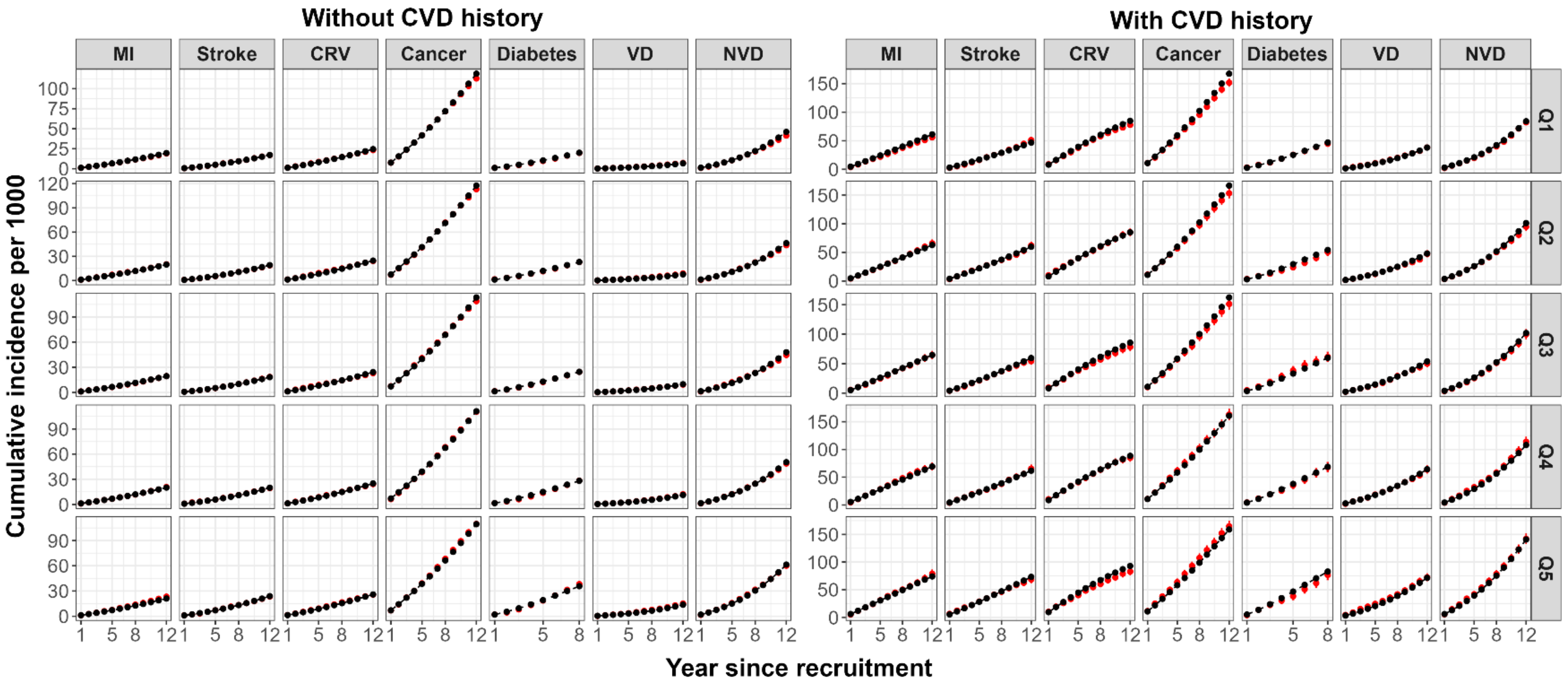

RED=Observed; BLACK=model. Validation includes three extra years that were not used to calibrate the model. MI, myocardial infarction; CRV, coronary revascularisation; VD, vascular death; NVD, non-vascular death. Follow-up of incident diabetes partly relies on primary care records, which ended earlier than other data types in the UKB. Incident diabetes is not predicted for individuals with diabetes history. Q1 (least deprived) to Q5 (most deprived), UK quintiles of socioeconomic deprivation.

Supplementary Figure S5: Accumulation of cardiovascular events of UKB participants over time in the model in following 30 years

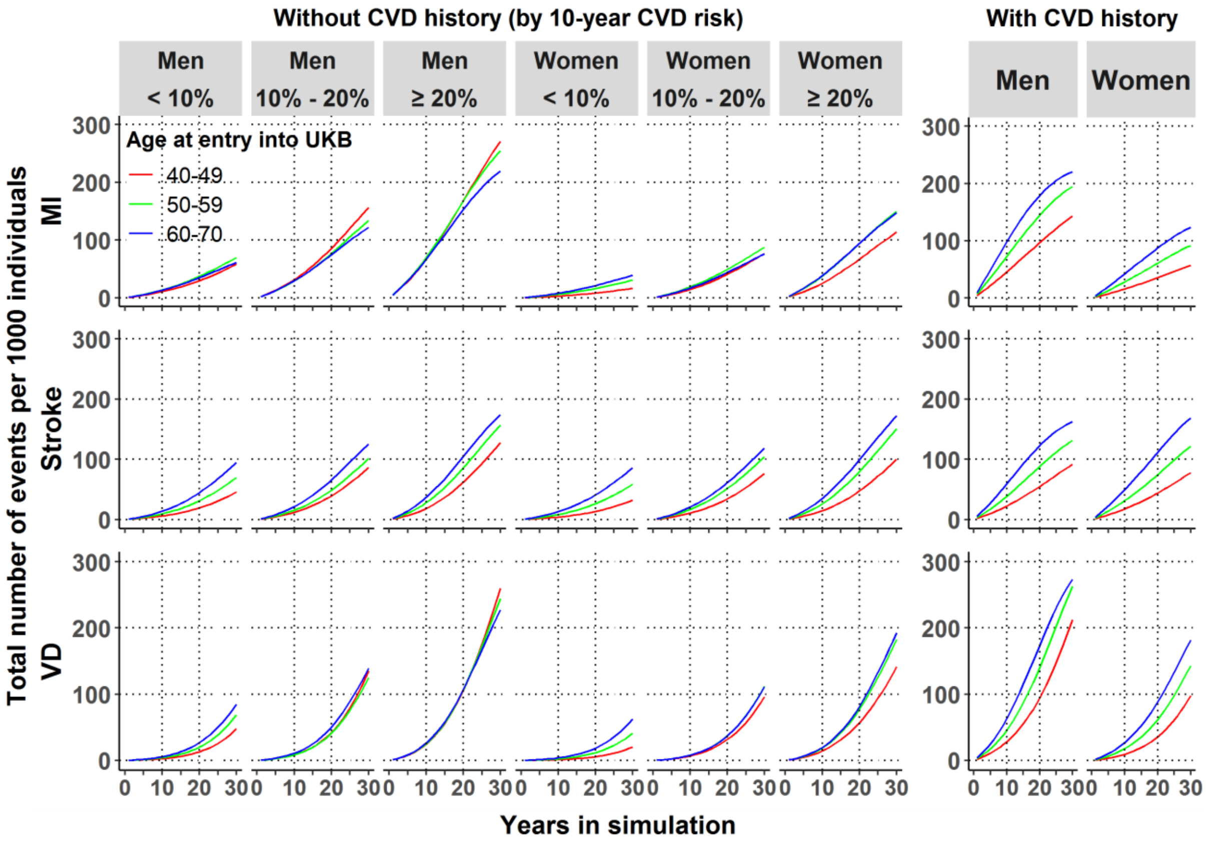

Predicted outcomes presented by CVD history, sex, age and, for people without CVD history, by 10-year CVD risk (QRISK3). UKB, UK Biobank; CVD, cardiovascular disease; MI, myocardial infarction; VD, vascular death.

Supplementary Figure S6: Predicted lifetime benefit from UK guideline-recommended statin therapy, by sex, age and quintile of socioeconomic deprivation in UK, with real-world statin use among eligible people

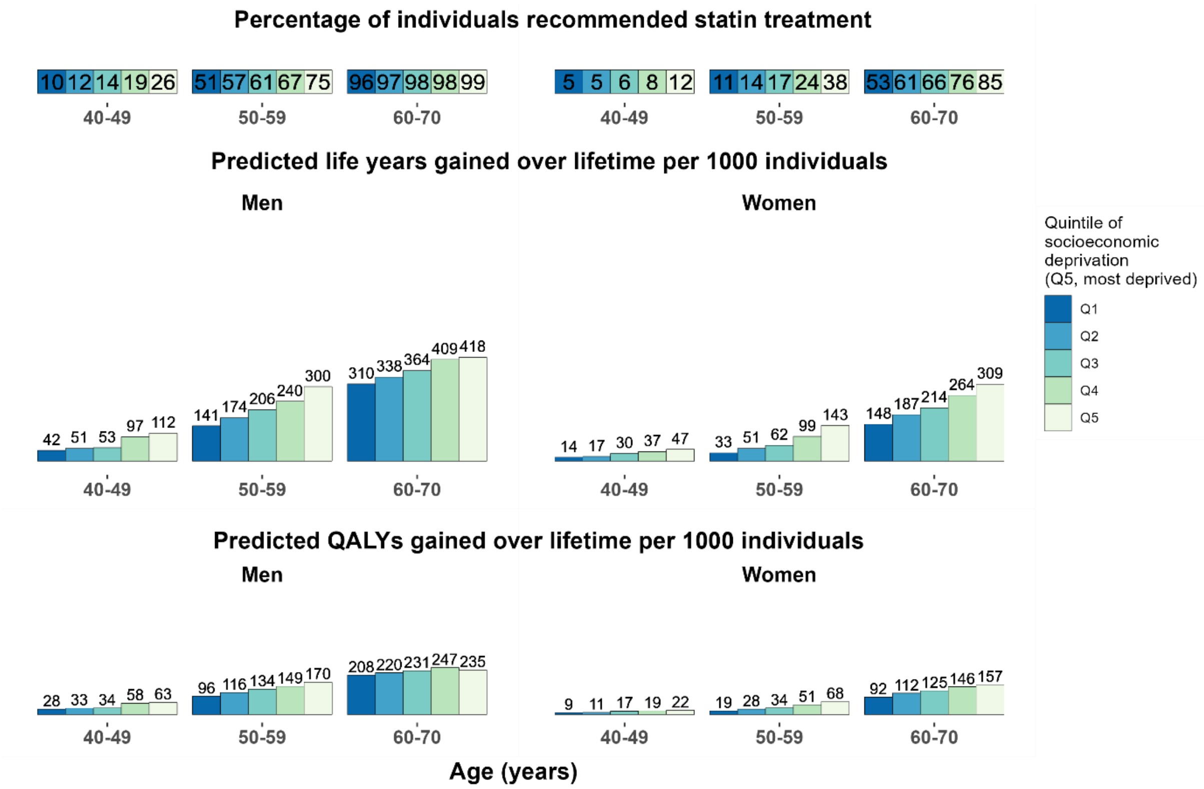

Predicted life years and QALYs gained with real-world statin use among eligible individuals, by age, sex, and socioeconomic deprivation quintiles (using Townsend score) at entry into UK Biobank. Results were standardised to mid-2020 UK population distribution by age, sex and quintile of socioeconomic deprivation. The percentages of individuals meeting the criteria for statin treatment in categories of UK Biobank participants are reported at the top. The bars represent predicted life years and QALYs gained per 1000 individuals, assuming the percentage of individuals recommended statin therapy in UK population categories by age, sex and socioeconomic deprivation is the same as in the corresponding categories in UK Biobank. Real-world statin use was informed by the rates of statin prescription between 1st June 2015 and 31st May 2016 among UKB participants with linked primary care records who were eligible for statin therapy according to the NICE guideline. NICE, National Institute for Health and Care; QALY, quality-adjusted life year.

Supplementary Figure S7: Model predicted 10-year risks of major vascular event versus QRISK 3 10-year CVD risk at baseline for UKB participant without previous cardiovascular disease at entry, by sex

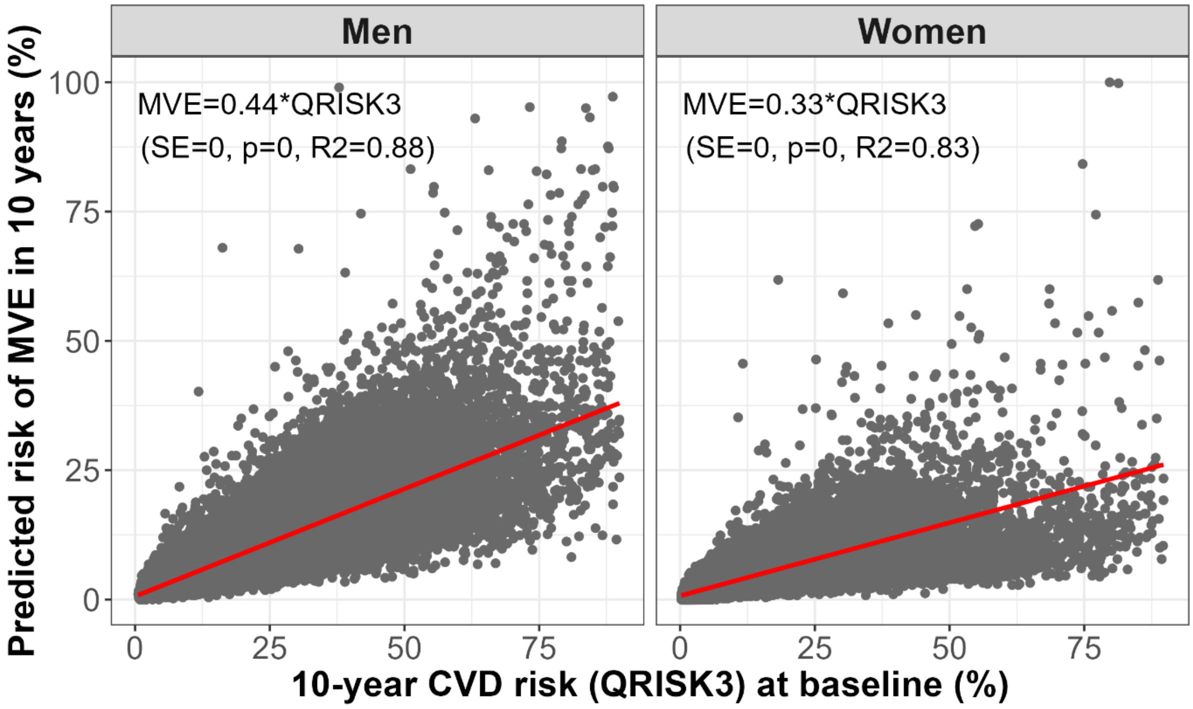

The 10-year risk of major vascular event (MVE, including MI, stroke, coronary revascularisation and vascular death) was predicted using the CVD microsimulation model. The 10-year CVD (i.e. coronary heart disease, ischaemic stroke, or transient ischaemic attack) risk was estimated using QRISK 3 risk prediction algorithms.(9)

Linear regressions, fit among men and women, suggest that the model predicted risks of MVE are highly correlated with QRISK3 scores with predicted MVE risk between 0.33 (women) and 0.44 (men) of QRISK3, respectively. UKB, UK Biobank; CVD, cardiovascular disease; MI, myocardial infarction.

Supplementary methods 1: Data preparation

The methods of handling missing data in UK Biobank

Following data identification and specification, several characteristics, such as ethnicity, smoking status, BMI, LDL cholesterol, HDL cholesterol, creatinine and blood pressure measures (and height, weight, cigarettes per day, Townsend score and total cholesterol for calculating QRISK3) had missing values (Supplementary method 1 table). Missing ethnicities were imputed as white, the majority ethnicity. Missing smoking statuses were imputed as the majority status in categories by sex, age groups and education levels. Missing cigarettes per day were imputed as the majority level, i.e. 10-20. Missing Townsend scores were imputed by regressing it on IMD scores (index of multiple deprivation), years and sources (England, Wales or Scotland) if IMD scores were available, and imputed by looking up an average Townsend score according to the rounded ordnance survey coordinates if IMD scores are missing, or imputed by looking up an average Townsend score for the areas of the assessment centres if IMD scores and ordnance survey coordinates were both missing. The remaining continuous variables with missing values were imputed using multiple imputation by chained equations with 20 imputations and 10 iterations for each using the package "mice" in R, with weight, height, LDL cholesterol, HDL cholesterol, triglycerides, creatinine, systolic blood pressure (two measures) and diastolic blood pressure (two measures), and with age, sex, (imputed) ethnicity, (imputed) smoking status, baseline cardiovascular diseases, treated hypertension, statin treatment status and diabetes added as auxiliary variables. After imputation, participants' QRISK3 scores were calculated with a published R package.(10)

Supplementary method 1 Table 1: Missing data in UK Biobank

	**Without CVD history N = 444,576**	**With CVD history N = 57,278**
	**Missing n (%)**	
Ethnicity	2411 (0.5%)	360 (0.6%)
Townsend score	553 (0.1%)	69 (0.1%)
Smoking	2480 (0.6%)	466 (0.8%)
Physical activity*	87,282 (19.6%)	12,714 (22.2%)
Diet quality	9482 (2.1%)	1536 (2.7%)
Body mass index	2457 (0.6%)	630 (1.1%)
LDL cholesterol	29,699 (6.7%)	4045 (7.1%)
HDL cholesterol	64,176 (14.4%)	8354 (14.6%)
Creatinine	29,130 (6.6%)	3962 (6.9%)
Systolic blood pressure	1155 (0.3%)	164 (0.3%)
Diastolic blood pressure	1153 (0.3%)	164 (0.3%)

*Missing values for physical activity level were not imputed but coded as a separate level. CVD, cardiovascular disease; LDL, low-density lipoprotein; HDL, high-density lipoprotein

QRISK3 derivation in UK Biobank

In addition to the characteristics previously defined for the CTT model and those explicitly derived from UKB, some assumptions had to be made to facilitate QRISK derivation. First, we assumed that all unspecified black people are black Caribbean. Second, we used the whole chronic renal failure category to substitute CKD stage 3-5 required by QRISK3, because at the baseline in UKB (2006-2010) few were coded as CKD stage 3-5 and many were coded as unspecified CKD. Third, we used the whole categories of depressive episode and recurrent depressive disorder to substitute moderate/severe depression required in QRISK3, because many were coded as unspecified depression in UKB. Fourth, we used history of heart disease of father, mother and sibling to substitute angina or heart attack in a 1st degree relative <60 required in QRISK3, as this is the closest information available in UKB. The code lists of medications for antihypertension, erectile dysfunction treatment, regular steroid tablets and atypical antipsychotic treatment referred to a published study using UKB data.(11)

Quintiles of Townsend score

According to UK Biobank website, "Townsend deprivation index calculated immediately prior to participant joining UK Biobank. Based on the preceding national census output areas. Each participant is assigned a score corresponding to the output area in which their postcode is located."(12) As the baseline assessment happened between 2006 and 2010, and Townsend indexes are published every ten years using the national census data, Townsend indexes for the baseline should be the 2001 version. With UK 2001 census data, we calculated Townsend indexes for all output areas in England and Wales using the method described by Paul Norman.(13) Then, we applied the England&Wales-wide quintile thresholds to the Townsend indexes to assign UK Biobank participants into the UK deprivation quintiles.

Definition of physical activity levels

The physical activity categories were based on International Physical Activity Questionnaire (IPAQ) with principles as follows.(14)

Supplementary method 1 Table 2: Definition of physical activity levels

Low physical activity	Moderate physical activity	High physical activity
no activity or not enough to meet moderate or high level	3 or more days of vigorous-intensity activity of at least 20 min/d **OR**<br>5 or more days of moderate-intensity activity and/or walking of at least 30 min/d **OR**<br>5 or more days of any combination of walking, moderate or vigorous-intensity activities achieving	vigorous-intensity activity on at least 3 days and accumulating at least 1500 MET-min/w **OR**<br>7 or more days of any combination of walking, moderate or vigorous-intensity activities achieving a minimum of at least 3000 MET-min/w

	a minimum of at least 600 MET-min/w	

The 20% of missing values were categorised into a missing category.

Definition of severe mental illness

The algorithm we used is based on a previous study,(15) and included the following ICD-10 codes: F20 Schizophrenia; F23 Acute and transient psychotic disorders; F31 Bipolar affective disorder; F32 Depressive episode; F33 Recurrent depressive disorder.

Definition of unhealthy diet

Daily diet was considered to be healthy if it met at least 4 of the following 7 components,(16) otherwise was considered “unhealthy”.

Supplementary method 1 Table 3 The components of healthy diet

**Component of food frequency**
Fruits: ≥ 3 servings/day
Vegetables: ≥ 3 servings/day
Fish: ≥2 servings/week
Processed meats: ≤ 1 serving/week
Unprocessed red meats: ≤ 1.5 servings/week
Whole grains: ≥ 3servings/day
Refined grains: ≤1.5servings/day

The 11018 (2%) uncertain cases due to missing data in some categories of food intake were combined with unhealthy diet, as early investigations suggested similarly sized associations for uncertain and unhealthy diets with endpoints’ risks.

Definition of diabetes refined using primary care data

The initial definition of diabetes employed the UKB algorithm (Table S1), which mapped the first occurrence of diagnostic codes across multiple information sources into the ICD 10 system with the earliest occurrence date as the diagnosis date. For diabetes diagnosis, Read C10E codes, ICD-10 E10 codes and self-reported Type 1 diabetes diagnosis were categorised into insulin dependent diabetes (Type 1); Read C10F family, ICD-10 E11 family and self-reported Type 2 diabetes diagnosis were categorised into non-insulin dependent diabetes (Type 2); other diabetes related diagnostic codes and self-reported unspecified diabetes were categorised into other specified or unspecified diabetes.

We adopted two further methods that were not used in the UKB algorithm.(17) The first is using medication information from the primary care records to detect diabetes among those without a diagnosis of diabetes. The second is using the timing of insulin use to identify Type 1 diabetes, in order to discriminate other specified or unspecified diabetes derived from the UKB algorithm.(17) The detail of the algorithm is presented in following table.

Supplementary method 1 Table 4: Definitions of diabetes types

	Type 1 diabetes	Type 2 diabetes
UKB algorithm alone	Insulin dependent diabetes*	Non-insulin dependent diabetes*
UKB algorithm + primary care records	Other specified or unspecified diabetes and using insulin <12 months post-diagnosis	other specified or unspecified diabetes, and not identified as Type 1 diabetes
Primary care records alone	Medication (insulin or non-metformin anti-diabetic drug) and using insulin <12 months post-recruitment	Medication (insulin or non-metformin anti-diabetic drug) and not identified as Type 1 diabetes

*If participants have ICD-10 E13/14 code but also have ICD-10 E10 or E11 code, they are coded as the respective type of diabetes, but the diagnosis date is updated to the earliest. Participants can be coded as Type 1 and Type 2 diabetes simultaneously.

Because the number of identified cases of Type 1 diabetes was small (less than 2%) relative to the total number of diabetes cases in the UKB, we did not split incident diabetes cases by types. Instead, we added an indicator of baseline Type 1 diabetes as a candidate covariate in risk equations of other endpoints for selection.

In addition, individuals without a diagnosis of diabetes and with HbA1c >= 48 mmol/mol were coded as with a diagnosis of diabetes one day before entry into the UKB study. We also integrated HbA1c into the diabetes history covariate in the other risk equations in the model. Specifically, the pre-diabetes status was categorised into four HbA1c strata, with continued use of diabetes duration alone to categorise post-diabetes diagnosis status (Figure 2 & Table S3-5).

Supplementary methods 2: The modelling procedure

The initial estimation of model risk equations

We initially estimated risk equations for six model endpoints, namely MI, stroke, coronary revascularisation, incident cancer, vascular and nonvascular death, based on the Cholesterol Treatment Trialists' (CTT) Collaboration IPD. At the time of this analysis, data on incident diabetes was not systematically available across trials. The CTT collaboration, described previously(18), has coordinated a prospective meta-analysis of all large statin trials cross multiple countries since 1990s. Briefly, the CTT database includes randomised trials that had lowering LDL cholesterol (LDL-C) as the main effect of an unconfounded trial intervention and aimed to recruit 1000 or more participants with a scheduled duration of follow-up of at least 2 years.(18) Individual participant data from 27 trials with 170,000 individuals were available in CTT database phase 1.(19) The present analysis included the CTT trials that were reported by the end of 2009 and provided data before June 2011 and excluded trials exclusively among patients suffering (end-stage) chronic kidney disease or chronic heart failure or comparing more versus less intensive statin therapy, and finally included 117,896 individuals from 16 trials.

The candidate predictor covariates included age, sex, ethnicity, body mass index (BMI), smoking status, blood pressure, lipids and creatinine levels, treated hypertension, previous CVD history, diabetes and cancer history. For each endpoint risk equation, other non-fatal model endpoints were included as annually updated covariates, with four categories initially (the same year as endpoint, one year ago, two year ago, three or more years ago), which were further combined through folding from the bottom if adjacent categories were not found to differ statistically at $p = 0.01$. All covariates of risk equations were selected in a backwards stepwise Cox semi-parametric models based on the likelihood ratio test with the inclusion threshold of $p = 0.01$. Proportional hazard assumption of covariates was tested using Schoenfeld Residuals for each Cox models. Parametric proportional hazards models with three distributions (exponential, Weibull and Gompertz) were fitted for each endpoint and their Akaike information criterion (AIC) and Bayesian information criterion (BIC) were compared to identify best fitting distributions. For risks of MI, stroke, coronary revascularisation, vascular death and nonvascular death, separate risk equations were estimated for participants without and with CVD history in acknowledgement of differences in associations between risk factors and event risks in these two populations. For incident cancer, a single risk equation was estimated across all participants as no such differences were observed between the two populations.

We coded the microsimulation model in R 4.2.1. Using the parametric risk equations derived from CTT, the microsimulation model annually simulates in a random order model events for an individual using their characteristics up to a pre-specified age or death. In each annual cycle, an individual can experience one or more model endpoints, and can die from either vascular or nonvascular cause. The experience of event/s influences future event risks as the risk equations include annually updated covariates for events. Consistent with estimated risk equations, the model simulates only first occurrences of each model event. We plotted model predicted cumulative risks of each event against observed risks to assess the internal performance of the model in categories of CTT participants.

Calibration of model risk equations and further development of the model

The risk equations were validated and calibrated in the UK Biobank (UKB) population. UKB is a large prospective cohort of more than 500,000 UK participants aged 40 to 70 years at recruitment between 2006 and 2010.(20) The UKB database provided detailed individual participant characteristics including demographic, behavioural, physical, and clinical characteristics and disease histories at recruitment. All UKB participants were linked to their national electronic records for death, cancer registry and hospital admission and about 45% of them are also linked to their primary care records for follow-ups. For the present analyses, all UKB participants with their linked data were included, except a small number of participants with end stage kidney disease.

We identified disease incidents using multiple sources in UKB to improve accuracy (Table S1). We used the UKB follow-up data up to 31st March 2017 for initial model calibration and further development, to ensure precise identifications of the first occurrence of model endpoints, because a few UKB data sources, such as hospital episodes statistics and cancer registry data, were available only until then at time of analysis.(21) The general strategy of model calibration/development in UKB was as follows:

1) calibrated the intercept and shape for all CTT-based risk equations using the linear predictor calculated using CTT risk equation coefficients and the corresponding UKB participant characteristics;

2) re-estimated coefficients of variables with a differing definition in UKB (smoking, ethnicity, diabetes and cancer) and related interaction terms by releasing them from the linear predictor;

3) included new covariates of interest that were not present in CTT (physically activity, socioeconomic deprivation, diet, mental illness and Type 1 diabetes); and

4) fitted an incident diabetes risk equation with hemoglobin A1c (HbA1c) as an extra covariate using data from UKB participants who have primary care records (a considerable number of incident diabetes were first detected in primary care only.

The incident diabetes risk equation was estimated across both participants with and without previous CVD history as there was no external evidence for difference in association with risk factors, the proportional hazards assumptions were met and there were no substantive differences in estimated associations in the two populations. The incident diabetes equation was integrated as an additional endpoint in the microsimulation model.

For the endpoints of death, cancer and diabetes, lifetime simulations were used to check if the model predicted lifetime risks were plausible compared with epidemiological studies' data and further. This indicated a need to adjust the incident cancer risk after 80 years of age as data in older people in UKB was very limited and failed to capture the reduced increase in cancer rate after age 75-80 and the peak in rate at 85-90 years of age.(22, 23) This adjustment used data from Cancer Research UK (CRUK),(22) Firstly, we used the CRUK data for people aged 40-79 years old to fit a simple linear regression model on incident cancer rates with a squared age and its interaction with sex as the predictors and the distribution of incident cancer cases by age of diagnosis in UKB as the weight. This model was to simulate the prediction of the UKB incident cancer risk equation (Model 1). Secondly, we fitted another simple linear regression model based on the CRUK data aged 50-90 with age splines to

capture the real pattern of incident cancer rates in the elderly (Model 2). Finally, we derived the ratios between the Model 1 predicted incident cancer rates against the Model 2 predicted incident cancer rates as the adjustment parameters, which were applied to adjust the transition probability of incident cancer predicted by the UKB incident cancer risk equation after 80 years of age.

Model simulation and summary of results

We used the CVD microsimulation model to perform lifelong projections for each participant in the UKB cohort. For participants on statin treatment at entry, their baseline LDL cholesterol levels were adjusted to pre-statin treatment level, using statin regimen information and statin efficacy data from systematic review of clinical studies.(24, 25) Individuals with cancer and/or diabetes history at entry were not at risk of incident cancer or diabetes in the model. We executed 500 model microsimulations for each individual. A round of simulation stops when the individual dies or reaches 110 years of age. Risks of model events, survival and QALY were summarised across the results of the 500 simulations by annual cycles, to minimise the Monte Carlo uncertainty in the model projection for each participant. The lifetime risks of model events, remaining life expectancy and QALYs for each participant were derived by adding up the results across all annual cycles, or across a certain number of annual cycles for a shorter-term projection. We summarised the results across participant categories by CVD history, age at entry, sex, 10-year CVD risk (QRISK 3 score)(9). We assessed parameter uncertainty in the model using the sets of bootstrap coefficients. We used 500 parameter sets for individual without CVD history (due to its large size, more rounds of simulation would take much additional time) and 1000 sets for individuals with CVD history.

Three extra years of follow-up data until 29th February 2020 (censored at this date to avoid influence from the COVID-19 pandemic), not used in the already completed model development, became available following release of further data from UKB and contributed to model validation. The only exception is primary care data, which was not available beyond 2016-2017 for the present study. Primary care data is required for the identification of incident diabetes and therefore, incident diabetes was validated over a shorter duration (Figures 2, S3 and S4).

Standardising model projections to UK population distribution

The model predicted life years and QALYs of UKB participants were summarised by single year of age, sex and socioeconomic deprivation quintiles, and further mapped to the UK population aged 40-70 in 2020 statistics (the combination of statistics from England, Wales, Scotland and Northern Ireland) across distribution of people by age, sex and socioeconomic deprivation quintiles (Index of Multiple Deprivation were used)(26-28). The English, Welsh and Scottish statistic tables were presented by single years of age, sex and deprivation deciles, and the Northern Irish statistic table were presented by 5-year age bands, sex and deprivation deciles. We converted deciles to quintiles and decomposed the Northern Irish statistic table into single years of age by referring to the distribution in the general population statistics of Northern Ireland in 2020.(28) To estimate the benefit of applying the preventive statin therapy recommended by the NICE guidance, we assumed that the proportions of individuals meeting the NICE criteria of using preventive statin therapy across the UK population categories by age, sex and socioeconomic deprivation are the same as the corresponding categories in the UKB cohort.

It should be noted that a key assumption in our approach to standardisation relies on the assumption that the UKB participant data by age, sex and socioeconomic deprivation is generalisable to the general UK population in these categories. While data within these categories are better able to be generalised, as confirmed by estimated gradients in risks across socioeconomic deprivation in some of the risk equations, we cannot be certain that we have comprehensively accounted for the differences between socioeconomic groups in the general population.

Supplementary methods 3: Description of Whitehall II data

Whitehall II is a cohort study among 10,308 participants aged 35-55 at the baseline, recruited from the British Civil Service in 1985, with periodic re-surveys of participants (called phases in Whitehall II). The Whitehall II study data includes Phases 1-9 and 11 at present. Due to attrition/no response and deaths, the numbers of participants declined from 10,308 in Phase 1 (1985-1988) to 6,308 in Phase 11 (2012-2014). During even-number phases, more limited baseline data was collected, and no biomarkers were measured. After weighing the data size and the length of follow-up, we used all participants of Phase 9 data to validate the CTT derived and UKB calibrated CVD model. Definitions and specifications are the same as data preparation in the UKB cohort data. The characteristics of Whitehall II Phase 9 participants are summarised in the following table.

Supplementary method 3 Table 1: Characteristics of Whitehall II Phase 9 participants (N = 6761)

	**Without prior CVD**	**With prior CVD**
**N**	**4,874**	**1,887**
	**Mean (SD) or count (%)**	
Age at entry at Phase 9 (years)	65.4 (5.8)	67.5 (6.1)
Male	3439 (71%)	1320 (70%)
Female	1435 (29%)	567 (30%)
Ethnicity		
White	4554 (93%)	1680 (89%)
South Asian	320 (7%)	207 (11%)
Smoking status		
Non-smoker	2601 (53%)	919 (49%)
Ex-smoker	1911 (39%)	811 (43%)
Current smoker	362 (7%)	157 (8%)
BMI (kg/m^2)	27 (4.4)	28 (4.7)
<18.5	49 (1%)	11 (1%)
18.5-25	1778 (36%)	523 (28%)
25-30	2080 (43%)	814 (43%)
30-35	757 (16%)	387 (21%)
35-40	162 (3%)	119 (6%)
40+	48 (1%)	33 (2%)
LDL (mmol/L)	3.1 (0.93)	2.8 (0.93)
On statin	1255 (26%)	
HDL (mmol/L)	1.6 (0.44)	1.6 (0.43)
Creatinine (umol/L)	84 (20)	89 (30)
Systolic BP (mmHg)	126 (16)	126 (17)
Diastolic BP (mmHg)	72 (10)	70 (10)
Baseline diabetes	667 (14%)	402 (21%)
Type 1 diabetes	3 (0%)	2 (0%)
Hypertension treatment	1404 (29%)	1111 (59%)
Baseline cancer	400 (8%)	161 (9%)
CVD history		
Other CHD only		1242 (66%)
MI only		97 (5%)

PAD only		90 (5%)
Stroke only		57 (3%)
Two or more		401 (21%)
Townsend score		
Quintile 1 (least deprived)		
Quintile 2	358 (7%)	112 (6%)
Quintile 3	3357 (69%)	1265 (67%)
Quintile 4	1157 (24%)	509 (27%)
Quintile 5	2 (0%)	1 (0%)
Physical activity*		
High level	1832 (38%)	613 (32%)
Moderate level	2829 (58%)	1138 (60%)
Low level	119 (2%)	77 (4%)
Missing	94 (2%)	59 (3%)
Severe mental illness	87 (2%)	39 (2%)
Unhealthy diet (incl. uncertain)	3051 (63%)	1192 (63%)

Electronic death records, NHS hospital records and cancer registry entries were linked for Whitehall II participants and used to identify events during follow-up. The data were made available until particular time points for different types of events. For incident coronary revascularisation and incident diabetes, only questionnaire data were available due to unavailable linked healthcare data, so we did not validate the two endpoints considering that the questionnaire data were unreliable. For the participants in Phase 9, deaths have the longest follow-up periods, with an average of 11.7 years, followed by MI and stroke with an average of 9.9 years. The follow-up periods of cancers are shorter, with an average of 6.1 years. The numbers of follow-up events for the older cohort are summarised in the following table.

Supplementary method 3 Table 2: Numbers of Whitehall II participants experiencing events following interviews in Phase 9

	**Myocardial infarction**	**Stroke**	**Incident Cancer**	**Vascular death**	**Nonvascular death**
**Without CVD history**	122	110	459	125	530
**With CVD history**	128	104	198	135	318
**Total**	250	214	657	260	848

Supplementary methods 4: Statin guideline recommendations and effects of statin treatment

Since 2014 the UK National Institute for Health and Care Excellence (NICE) guideline recommends atorvastatin 20mg daily to individuals without CVD history but with a 10-year CVD risk⩾10%, and/or type 1 diabetes, an estimated glomerular filtration rate (eGFR)<60mL/min/1.73^2, or albuminuria, and atorvastatin 80mg daily to individuals with CVD history (20mg to those with eGFR<60mL/min/1.73^2).(29)

Statin effects on cardiovascular endpoints

The effects of statin treatment were implemented in the model through reductions in risks of myocardial infarction, stroke, coronary revascularisation and vascular death, with sizes of risk reductions depending on LDL cholesterol reductions. We used the CVD microsimulation model to project event risks and survival and summarised life years and QALYs over individuals' remaining lifetimes (until death or reaching 110 years of age) without statin therapy and with full compliance of statin therapy among those UKB participants recommended for statin treatment by NICE.(29)

The following two tables present model parameters for proportional reductions in LDL cholesterol with different statin regimens, and the effects of statin therapy on cardiovascular events per 1 mmol/L reduction in LDL cholesterol, respectively. Atorvastatin 20mg and 80mg regimens were used in the illustration of the model application.

	**% reduction in LDL cholesterol with statin therapy***				
**Dose (mg/day)**	**5mg**	**10mg**	**20mg**	**40mg**	**80mg**
Fluvastatin	10%	15%	21%	27%	33%
Pravastatin	15%	20%	24%	29%	33%
Simvastatin	23%	27%	32%	37%	42%
Atorvastatin	31%	37%	43%	49%	55%
Rosuvastatin	38%	43%	48%	53%	58%

*NICE clinical guideline CG181 Lipid modification guideline, 2014, Table 36, page 126.(29)

	**Relative Risk (95% confidence interval) per 1mmol/L reduction in LDL cholesterol with statin therapy***
Myocardial infarction	0.76 (0.73-0.79)
Stroke	0.84 (0.80-0.89)
Coronary revascularisation	0.75 (0.73-0.78)
Vascular death	0.88 (0.85-0.91)

*Cholesterol Treatment Trialists' Collaboration individual participant data meta-analysis(19)

Adverse effects of statin treatment

The model takes into account adverse effects of statin treatment including excess incident diabetes, myopathy and rhabdomyolysis reported in the literature.(30) The following table presents the model parameters for the adverse effects.

**Incident diabetes**	
Standard statin therapy compared to no statin treatment*	Odds Ratio (95% CI) 1.09 (1.02-1.17)(31)
High intensity statin therapy compared to standard statin therapy#	Odds Ratio (95% CI) 1.12 (1.04-1.22)(32)
**Myopathy**	
Excess cases (95% CI) per 100,000 treated with statin therapy per year	11 (4-27) cases(33)
Reduction in quality of life over 30 days.	0.017 QALY reduction in year(34)
**Rhabdomyolysis**	
Excess cases (95% CI) per 100,000 treated with statin therapy	3.4 (1.6-6.5) cases(33)
Case fatality of rhabdomyolysis	10%(33)
Reduction in quality of life	50% over 7.5 days and 20% for further 30 days(34)

*standard statin therapy refers to statin regimen associated with proportional LDL cholesterol reduction of 35%-45%; #high intensity statin therapy refers to statin regimen associated with proportional LDL cholesterol reduction of 45% or higher.

Real-world statin use scenario

We simulated another scenario which took into account the effect of the real-world use of statin therapy in categories by sex, age and socioeconomic deprivation. We calculated the percentages of participants with statin prescription between 1st June 2015 and 31st May 2016 among UKB participants with linked primary care records who were eligible for statin treatment according to the NICE guideline(29) (see table below). In total, 151,161 participants (132,402 without and 18,759 with CVD history) had primary care records during this period. Among them, 75,190 participants (56,431 without CVD history and the 18,759 with CVD history) were eligible for statin treatment, and 29,736 (21,393 and 8,343, respectively) had records of statin prescription. Across sex and age, the statin prescription rates were 40%, 40%, 41%, 43% and 45% among the eligible patients from the least deprived quintile to the most deprived quintile, respectively.

Sex	Age	Townsend score quintile*	N†	Statin prescription rate
Men	40-49	Q1	547	33%
Men	40-49	Q2	356	30%
Men	40-49	Q3	412	30%
Men	40-49	Q4	545	39%
Men	40-49	Q5	625	38%
Men	50-59	Q1	4,211	36%
Men	50-59	Q2	2,590	37%
Men	50-59	Q3	2,145	39%
Men	50-59	Q4	2,105	39%
Men	50-59	Q5	1,988	44%
Men	60-70	Q1	12,204	46%
Men	60-70	Q2	6,445	46%
Men	60-70	Q3	4,571	47%

Men	60-70	Q4	3,940	51%
Men	60-70	Q5	2,941	53%
Women	40-49	Q1	347	15%
Women	40-49	Q2	223	14%
Women	40-49	Q3	216	25%
Women	40-49	Q4	293	29%
Women	40-49	Q5	341	33%
Women	50-59	Q1	1,258	29%
Women	50-59	Q2	929	34%
Women	50-59	Q3	910	34%
Women	50-59	Q4	1,072	39%
Women	50-59	Q5	1,198	41%
Women	60-70	Q1	7,585	36%
Women	60-70	Q2	4,788	38%
Women	60-70	Q3	3,854	37%
Women	60-70	Q4	3,650	40%
Women	60-70	Q5	2,901	42%

*Q5 is the most deprived quintile; †numbers of UK participants with primary care records in the analytic period who were eligible for statin treatment.

Supplementary references

1. NHS Connecting for Health Department of Health Social Care Information Centre. OPCS Classifications of Interventions and Procedures: The Stationery Office; 2006.
2. Wu M, Brazier JE, Kearns B, et al. Examining the impact of 11 long-standing health conditions on health-related quality of life using the EQ-5D in a general population sample. Eur J Health Econ. 2015;16(2):141-51.
3. Tsiplova K, Pullenayegum E, Cooke T, et al. EQ-5D-derived health utilities and minimally important differences for chronic health conditions: 2011 Commonwealth Fund Survey of Sicker Adults in Canada. Qual Life Res. 2016;25(12):3009-16.
4. Borchert K, Jacob C, Wetzel N, et al. Application study of the EQ-5D-5L in oncology: linking self-reported quality of life of patients with advanced or metastatic colorectal cancer to clinical data from a German tumor registry. Health Econ Rev. 2020;10(1):40.
5. Keng MJ, Leal J, Bowman L, et al. Decrements in health-related quality of life associated with adverse events in people with diabetes. Diabetes Obes Metab. 2021.
6. Geessink N, Schoon Y, van Goor H, et al. Frailty and quality of life among older people with and without a cancer diagnosis: Findings from TOPICS-MDS. PLoS One. 2017;12(12):e0189648.
7. Kroep S, Chuang L-H, Cohen A, et al. The impact of co-morbidity on the disease burden of VTE. J Thromb Thrombolysis. 2018;46(4):507-15.
8. Krishnan A, Teixeira-Pinto A, Lim WH, et al. Health-related quality of life in people across the spectrum of CKD. Kidney Int Rep. 2020;5(12):2264-74.
9. Hippisley-Cox J, Coupland C, Brindle P. Development and validation of QRISK3 risk prediction algorithms to estimate future risk of cardiovascular disease: prospective cohort study. BMJ. 2017;357:j2099.
10. Li Y, Sperrin M, van Staa T. R package “QRISK3”: an unofficial research purposed implementation of ClinRisk’s QRISK3 algorithm into R. F1000Research. 2020;8(2139):2139.
11. Carter AR, Gill D, Morris R, et al. Educational inequalities in statin treatment for preventing cardiovascular disease: cross-sectional analysis of UK Biobank. medRxiv. 2020.
12. UK Biobank. Description: Townsend deprivation index at recruitment. 24/08/2022. Available from: https://biobank.ndph.ox.ac.uk/showcase/field.cgi?id=189.
13. Norman P. Identifying change over time in small area socio-economic deprivation. Appl Spat Anal Policy. 2010;3(2):107-38.
14. UK Biobank. Guidelines for data processing and analysis of the International Physical Activity Questionnaire (IPAQ). 2005 24/08/2022. Available from: https://biobank.ndph.ox.ac.uk/showcase/ukb/docs/ipaq_analysis.pdf.
15. Carter AR, Gill D, Smith GD, et al. Cross-sectional analysis of educational inequalities in primary prevention statin use in UK Biobank. Heart. 2022;108(7):536-42.
16. Lourida I, Hannon E, Littlejohns TJ, et al. Association of lifestyle and genetic risk with incidence of dementia. JAMA. 2019;322(5):430-7.
17. Eastwood SV, Mathur R, Atkinson M, et al. Algorithms for the capture and adjudication of prevalent and incident diabetes in UK Biobank. PloS One. 2016;11(9):e0162388.
18. Downs J, Gotto A, Clearfield M, et al. Protocol for a prospective collaborative overview of all current and planned randomized trials of cholesterol treatment regimens. Am J Cardiol. 1995;75(16).
19. Cholesterol Treatment Trialists' Collaboration. Efficacy and safety of statin therapy in older people: a meta-analysis of individual participant data from 28 randomised controlled trials. Lancet. 2019;393(10170):407-15.
20. Sudlow C, Gallacher J, Allen N, et al. UK Biobank: an open access resource for identifying the causes of a wide range of complex diseases of middle and old age. PLoS Med. 2015;12(3):e1001779.
21. UK Biobank. Data providers and dates of data availability. 31/07/2023. Available from: https://biobank.ndph.ox.ac.uk/showcase/exinfo.cgi?src=Data_providers_and_dates.
22. Cancer Research UK. Cancer incidence by age2021 10/02/2022. Available from: https://www.cancerresearchuk.org/health-professional/cancer-statistics/incidence/age.

23. Harding C, Pompei F, Wilson R. Peak and decline in cancer incidence, mortality, and prevalence at old ages. Cancer. 2012;118(5):1371-86.
24. Law MR, Wald NJ, Rudnicka A. Quantifying effect of statins on low density lipoprotein cholesterol, ischaemic heart disease, and stroke: systematic review and meta-analysis. BMJ. 2003;326(7404):1423.
25. Wu R, Rison SCG, Raisi-Estabragh Z, et al. Gaps in antihypertensive and statin treatments and benefits of optimisation: a modelling study in a 1 million ethnically diverse urban population in UK. BMJ Open. 2021;11(12):e052884.
26. Office for National Statistics. Populations by Index of Multiple Deprivation (IMD) decile, England and Wales, 20202021 26/12/2022. Available from: https://www.ons.gov.uk/peoplepopulationandcommunity/populationandmigration/populationestimates/adhocs/13773populationsbyindexofmultipledeprivationimddecileenglandandwales2020.
27. National Records of Scotland. Population estimates by Scottish Index of Multiple Deprivation (SIMD)2022 24/12/2022. Available from: https://www.nrscotland.gov.uk/statistics-and-data/statistics/statistics-by-theme/population/population-estimates/2011-based-special-area-population-estimates/population-estimates-by-simd-2016.
28. Northern Ireland Statistics and Research Agency. 2020 mid year population estimates for Northern Ireland2021 24/12/2022. Available from: https://www.nisra.gov.uk/publications/2020-mid-year-population-estimates-northern-ireland.
29. National Institute for Health and Care Excellence. Lipid modification: cardiovascular risk assessment and the modification of blood lipids for the primary and secondary prevention of cardiovascular disease. 2014 27/05/2023. Available from: https://www.nice.org.uk/guidance/cg181/evidence/lipid-modification-update-full-guideline-243786637.
30. Collins R, Reith C, Emberson J, et al. Interpretation of the evidence for the efficacy and safety of statin therapy. Lancet. 2016;388(10059):2532-61.
31. Sattar N, Preiss D, Murray HM, et al. Statins and risk of incident diabetes: a collaborative meta-analysis of randomised statin trials. The Lancet. 2010;375(9716):735-42.
32. Preiss D, Seshasai SRK, Welsh P, et al. Risk of incident diabetes with intensive-dose compared with moderate-dose statin therapy: a meta-analysis. JAMA. 2011;305(24):2556-64.
33. Law M, Rudnicka AR. Statin safety: a systematic review. The American journal of cardiology. 2006;97(8):S52-S60.
34. Lazar LD, Pletcher MJ, Coxson PG, et al. Cost-effectiveness of statin therapy for primary prevention in a low-cost statin era. Circulation. 2011;124(2):146-53.
